# Rediscovery of the Lost and Critically Endangered Dehradun Stream Frog, *Amolops chakrataensis*

**DOI:** 10.3390/ani16193072

**Published:** 2026-09-29

**Authors:** Vishal Kumar Prasad, Saurav Chaudhary, Sky T. Button, Sumbul Gill, Amit Badola, Devendra Singh, Sandeep Kumar Gupta, K. P. Dinesh, Amaël Borzée

**Affiliations:** 1 Laboratory of Animal Behaviour and Conservation, College of Ecology and Environment, Nanjing Forestry University, Nanjing 210037, China; 2 Wildlife Institute of India, Dehradun 248001, Uttarakhand, India; sauravchaudhary533@gmail.com (S.C.); devurawat.dr@gmail.com (D.S.); skg@wii.gov.in (S.K.G.); 3 Amphibian Specialist Group, Species Survival Commission, International Union for the Conservation of Nature, 1196 Gland, Switzerland; 4 Laboratory of Animal Behaviour and Conservation, College of Life Sciences, Nanjing Forestry University, Nanjing 210037, China; sky.tc.button@gmail.com (S.T.B.); sumbulgil067@gmail.com (S.G.); 5 Biodiversity Research and Conservation Foundation, Dehradun 248001, Uttarakhand, India; amitbadola99@gmail.com; 6 Zoological Survey of India, Western Regional Centre, Pune 411007, Maharashtra, India; kpdinesh.zsi2@gmail.com; 7 Department of Zoology and General Biology, Faculty of Life Sciences, Fergana State University, Fergana 150100, Uzbekistan

**Keywords:** amphibian conservation, field surveys, habitat envelope, species rediscovery, threatened amphibians, western Himalayas

## Abstract

The Dehradun Stream Frog, *Amolops chakrataensis,* is a Critically Endangered stream-dwelling amphibian endemic to Uttarakhand in the western Himalayas of India and the species was previously known only from a single holotype collected in 1985. Despite repeated surveys, the species remained undetected for more than four decades, raising concerns that it went extinct. Here, we report the rediscovery of *A. chakrataensis* from the Maneri landscape, Uttarakhand, approximately 65 km on a straight-line east of the type locality. We confirmed the species identity using an integrative taxonomic approach combining morphological comparisons with the holotype, and we sequenced the mitochondrial 16S rRNA DNA gene fragment. Our analyses recovered *A. chakrataensis* as a distinct clade. Extensive field surveys covering 14 sites and 424 person-hours detected only two individuals, highlighting the rarity of the species. Using environmental characteristics of the three known localities, we developed a habitat envelope that identified additional priority areas for future surveys and potential conservation management. Our findings confirm the continued persistence of *A. chakrataensis*, provide the first molecular reference for the species, extend its known distribution, and establish an important foundation for future monitoring and conservation planning in the western Himalayas.

## Simple Summary

We documented the rediscovery of the extremely rare and Critically Endangered *Amolops chakrataensis* in the Maneri landscape, Uttarkashi district in Uttarakhand, approximately 65 km from its type locality, representing only the second confirmed record of the species since its original description in 1992. Morphological comparisons confirmed the species identity of the individual, and we generated the first mitochondrial 16S rRNA sequence for the species. The rediscovery extends its known distribution and provides crucial baseline information for future monitoring and conservation. Using the environmental characteristics of the three known localities, we also identified additional areas with suitable habitat, highlighting priority sites for future surveys and conservation actions. These findings improve our understanding of the species’ current distribution, provide the first molecular reference for reliable identification, and offer a practical framework for locating remaining populations and guiding conservation efforts in the western Himalayas.

## 1. Introduction

Amphibians with rare and restricted distributions are particularly vulnerable, as even localised habitat degradation can rapidly drive population declines and increase extinction risks [1,2]. The combined effects of these threats, magnified by increasing climate warming, are expected to exert additional pressure on the narrowly distributed Himalayan biodiversity, including amphibians that depend on high-altitude, perennial mountain streams [3,4,5]. Rediscoveries of narrowly distributed species are therefore of high conservation importance because they confirm persistence, refine known distributions, and provide an opportunity to reassess conservation priorities using contemporary field data [6,7,8]. This is particularly relevant for stream-dwelling amphibians, whose populations are highly dependent on the integrity of freshwater ecosystems.

The Dehradun Stream Frog, *Amolops chakrataensis,* is one of India’s rarest amphibians, and is endemic to the western Himalayas of Uttarakhand [9,10,11]. This species was described from a single specimen collected near Chakrata, Uttarakhand, in 1985, and remained one of the least-known members of the genus for more than four decades [9,12]. Owing to its known restricted distribution, with the species known from at a single threat-defined location (sensu IUCN) with declining habitat quality, and because of continued declines in habitat quality, *A. chakrataensis* is currently listed as Critically Endangered on the IUCN Red List [10]. Despite its conservation significance, nothing is known about its population size, ecology, breeding biology, or complete distribution, limiting effective conservation planning. Intensive field surveys in the past across the type locality and surrounding streams have failed to detect *A. chakrataensis* [11], despite false hopes following a misidentification in 2011 [13]. As a result, the species remained listed as Data Deficient by the IUCN Red List until 2023 [10,14]. The absence of confirmed presence raised concerns that the species became locally extirpated from its only confirmed locality in Chakrata [11]. At the same time, these findings also emphasised the urgent need for expanded surveys beyond the type locality.

Here, we report the rediscovery of *A. chakrataensis* after 41 years of sightings. We confirm the identity of the species using morphological comparisons with the holotype and mitochondrial 16S DNA sequences and further identify additional areas of potential occurrence using a habitat-envelope model.

## 2. Materials and Methods

### 2.1. Sampling and Data Collection

We carried out field surveys for *Amolops chakrataensis* Ray, 1992, from 2023 to 2026. Our sampling covered amphibian peak activity seasons in the western Himalayas: spring (February–March), summer (April–May), monsoon (June), and post-monsoon (September–October). *Amolops* frogs are most active during these times [11,12,15]. We planned our surveys to match periods when snowmelt-fed Himalayan streams provide suitable conditions for amphibian activity and breeding. We avoided the extreme cold season, when amphibians hibernate in the western Himalayas, and the peak monsoon, when high water flow and levels cause floods and reduce the stable stream habitats needed for breeding and egg-laying [11,15]. Trained researchers with amphibian identification experience conducted the surveys.

We conducted daytime (1030–1600 h) and nighttime (1800–2330 h) visual encounter surveys, along with aural surveys, following established protocols [15,16,17,18]. We surveyed each site at least twice; Supplementary Table S1 provides details of person-hours at each site. We surveyed Tuna Forest, Masak, Jadi Village, Kanaser, Lokhandi Village, Moila Forest, Gvasa Forest, Tiger Waterfall, Dava, Dabri Khadd, Sahiya, and four sites within the Maneri landscape, spanning elevations of approximately 1100 m–2400 m a.s.l. within an approximately 100 km radius around the type locality in Uttarakhand. Details of surveys sites are provided in Supplementary Table S1. We focused primarily on small- to medium-sized headwater streams, from single isolated streams a few metres wide, to 50 m wide riverbeds with several parallel streams, but all are characterised by boulders emerging above the water.

We followed a standardised survey approach across all sites. At each locality, we surveyed approximately 1 km of stream over 4 h for each sampling day, within suitable habitat for *Amolops* species, characterised by fast-flowing water and boulders. We split the area we surveyed into two equal transects per stream, systematically searching stream sections of approximately 500 m in length for 2 h each, allowing for the careful inspection of potential microhabitats.

We encountered two individuals of *A. chakrataensis*. We captured one individual, but the second escaped. We collected a DNA sample using non-invasive buccal swabs for genetic analyses [19]. We collected the buccal swab in the field at Maneri and stored the swab in a 2 mL tube containing a silica desiccant bead for 10 days before transferring it to a −20 °C freezer at the Laboratory in the Wildlife Institute of India.

We followed standard amphibian biosecurity protocols by wearing disposable gloves while handling amphibians and sterilising field equipment and footwear between survey sites to minimise the risk of pathogen transmission throughout the surveys [20].

### 2.2. Morphology

We photographed a single successfully captured *A. chakrataensis* individual using a DSLR camera (Nikon, Tokyo, Japan) and measured the morphological characters of the live individual in the field using digital callipers (CD-ASX, Mitutoyo, Kawasaki, Japan). To ensure accurate measurements and minimise bias, the individual was placed on a damp, non-slip surface, and we allowed the individual to settle for 10 s before taking the measurements. A researcher wearing clean disposable gloves gently restrained the frog by supporting the body and limbs while another researcher recorded the measurements. Handling of the animal followed animal welfare guidelines of the Uttarakhand Forest Department and the IUCN recommendations for sampling of species at risk of extinction [21].

The holotype of *A. chakrataensis* was examined at the Zoological Survey of India collections in Dehradun (NRS A-16, ZSI–NRC). For both the live individual and the holotype museum specimen, we measured the same set of 11 morphological characters to the nearest 0.1 mm using a Mitutoyo digital calliper (CD-ASX, Mitutoyo, Kawasaki, Japan): snout–vent length (SVL), from the tip of the snout to the vent; head length (HL), from the back of the mandible to the tip of the snout; head width (HW), measured at the jaw angle; eye diameter (ED), measured horizontally between the anterior and posterior margins of the eye; tympanum diameter (TD), maximum horizontal width; forelimb length (FLL), from the elbow to the base of the outer palmar tubercle; hand length (HAL), from the base of the outer palmar tubercle to the tip of the third finger; tibia length (TL), from the outer surface of the flexed knee to the tibiotarsal inflexion; femur length (FL), from the vent to the knee; foot length (FOL), from the base of the inner metatarsal tubercle to the tip of the fourth toe; and inner metatarsal tubercle length (IMT). For the newly recorded individual only, we additionally measured snout length (SL), internarial distance (IND), and interorbital distance (IOD). We diagnosed morphological characters from the literature for the following *Amolops* [22]. We followed the taxonomy of [23].

### 2.3. DNA Barcoding

We extracted Genomic DNA using the DNeasy Blood and Tissue Kit (Qiagen, Hilden, Germany) following the manufacturer’s protocol. Both forward and reverse strands of a fragment of the mitochondrial 16S rRNA gene were sequenced. We amplified a fragment of the mitochondrial 16S rRNA gene using universal primers [24]. The raw forward and reverse chromatograms were trimmed, manually inspected for sequencing errors, and assembled into a 599-bp consensus sequence using Geneious Prime v. 2023.2.1 [25]. The sequence generated in this study was deposited in GenBank under accession number (awaiting from GeneBank; Supplementary Table S2). To confirm species identity, the newly generated 16S mtDNA sequence was aligned with 260 homologous 16S sequences of *Amolops* species retrieved from GenBank (Supplementary Table S2). We selected four species, *Nidirana hainanensis*, *Rana jiemuxiensis*, *Odorrana jingdongensis*, and *Huia cavitympanum*, as outgroups (Supplementary Table S2). 

The best-fit nucleotide substitution model for the 16S dataset was selected using PartitionFinder v. 2.1.1 [26] by adjusting the settings: branch lengths unlinked, corrected Akaike information criterion (AICc), and greedy search algorithm [26], with GTR + I + G identified as the optimal model. Maximum-likelihood (PhyML) served as a methodological cross-check, allowing for comparison of topologies derived under the Bayesian framework [26,27,28]. Bayesian phylogenetic analyses were conducted using MrBayes v. 3.2.7 [29] with four Markov chain Monte Carlo (MCMC) chains, each ran for 10,000,000 generations. Trees were sampled every 1000 generations, and the first 25% of sampled trees were discarded as burn-in [29]. Convergence was assessed by ensuring that the average standard deviation of split frequencies was <0.01. Tracer v1.7.2 was used for examining parameter traces, and effective sample sizes (ESS) for all parameters achieved ESS values > 200, indicating adequate sampling from the posterior distribution [30]. Uncorrected pairwise genetic distances (p-distances) were calculated in MEGA X [31] to quantify genetic divergence among taxa. The resulting phylogeny was used to confirm the taxonomic placement and species identity of *A. chakrataensis*.

### 2.4. Potential Habitat Approximation

To approximate the potential habitat for *A. chakrataensis*, we used a simple habitat-envelope-based approach, leveraging key widespread habitat associations for the genus *Amolops* in general [11,12,15]. These analyses were carried out within a 100 km buffer of known *A. chakrataensis* localities, including the type locality and locations newly discovered in this study. Importantly, additional sampling and occurrence points will be needed to construct more robust distributional models [32]. However, simpler envelope-based approaches can nonetheless help guide future surveys and initial management frameworks for species lacking extensive data [33]. To implement this approach, we used five variables with which *A. chakrataensis* tend to have strong associations, including elevation, flow accumulation (as a proxy for watercourse size), average slope within 300 m, percentage forest cover within 1 km as of 2020 [34], and dominant lithology type [35]. Three occurrence points (one from the original type locality; two representing newly discovered populations) were used to infer approximate habitat associations within this framework. For quantitative variables (i.e., all except forest cover), we approximated potentially suitable ranges as being bounded by the lowest and highest values across the species’ three occurrence points, plus a 10% buffer to account for uncertainty and poor sample size. However, as *A. chakrataensis* is most likely associated with forested streams with boulder-dominated substrates [11,12], consistent with the ecology reported for closely related congeners in the Himalayas [12,15,36], we did not set an upper boundary on the acceptable range for this variable. Next, we calculated areas where all four variables fell within these buffered ranges, then computed the subset matching the dominant lithology type for at least one existing locality. Finally, we mapped a further subset of these areas by erasing all areas within a 1 km buffer of existing amphibian survey efforts failing to document the species (inferred from a combination of Global Biodiversity Information Facility [37] amphibian records and surveys conducted in this study). The remaining areas were treated as higher priorities, based on closely matching habitats and a lack of any previous surveys discounting *A. chakrataensis’* presence. While unlikely to accurately capture the nuances of *A. chakrataensis’* habitat requirements, these final areas represent the highest priorities for future surveys and tentative species management given the limited information currently available.

## 3. Results

Our study provides evidence for the rediscovery of *Amolops chakrataensis* after approximately four decades since its last confirmed record, represented by the collection of the holotype in 1985 [9,11,12].

### 3.1. Surveys

We spent 424 total person-hours surveying our 14 total study sites, resulting in one captured and another detected *A. chakrataensis* at two new localities, located approximately 65 km east of the type locality. Thus, *A. chakrataensis* was detected at a rate of only one individual per 212 person-hours of survey effort (Supplementary Table S1).

### 3.2. Morphology

**Figure 1:**
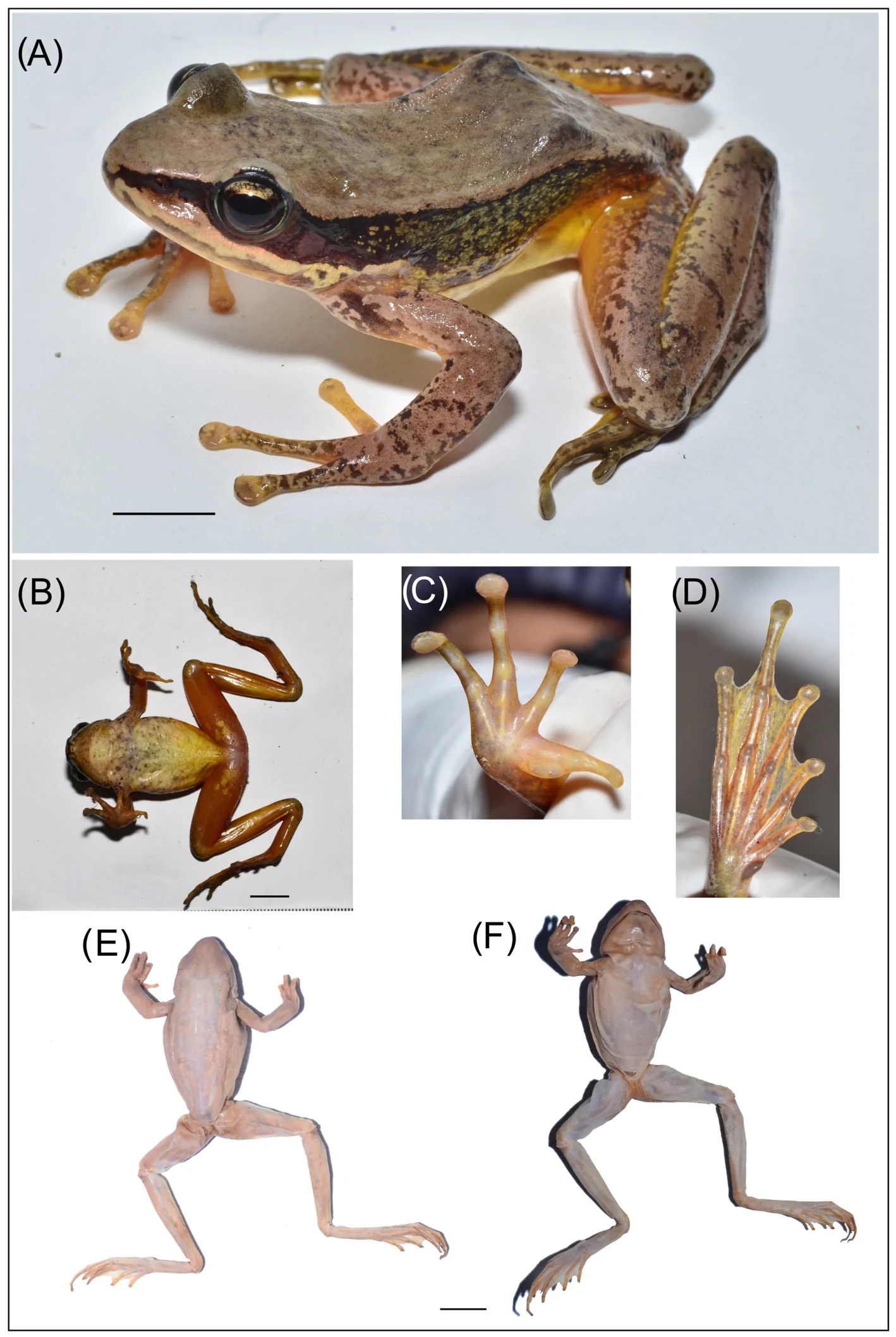
Photographs of the rediscovered *Amolops chakrataensis* from the Maneri landscape, Uttarakhand, India. (**A**) Dorsolateral view of the live individual showing the overall body shape and colouration in life. (**B**) Ventral view of the live individual showing ventral colouration. (**C**) Ventral view of the hand of live individual. (**D**) Ventral view of the foot of live individual. (**E**) Dorsal view of the preserved holotype (NRS A-16, Zoological Survey of India, Northern Regional Centre, Dehradun). (**F**) Ventral view of the preserved holotype. Scale bars = 10 mm. Photographs: © (**A**–**D**), Saurav Chaudhary and Amit Badola; © (**E**,**F**), Vishal Kumar Prasad.

All morphological characters matched the original descriptions of *A. chakrataensis* [9,12]. The examined individual was a medium-sized adult (SVL 58.1 mm; Table 1; Figure 1). The head was dorsoventrally flattened and wider than long. The snout was obtusely pointed in dorsal view and projected beyond the lower jaw. The interorbital distance exceeded the upper eyelid width. A pineal ocellus was visible on the dorsal surface between the anterior margins of the orbits. The canthus rostralis was well defined, and the loreal region was concave. The eyes were moderately large. A prominent supratympanic fold extended from the posterior corner of the eye posteroventrally towards the shoulder, concealing the tympanum, which was approximately three-fifths of the eye diameter. The dorsum was flattened with a gentle anterior slope, and the dorsal skin was smooth to shagreened. Two prominent dorsolateral glandular folds originated behind the upper eyelids and extended posterodorsally to the sacral region. The flanks were glandular and covered with numerous minute yellowish glandular granules. The forelimbs were muscular, with long, slender fingers bearing expanded discs; relative finger lengths were F1 < F2 < F4 < F3. The hind limbs were moderately long, with toes bearing expanded discs that were smaller than those of the fingers and were extensively webbed; relative toe lengths were T1 < T2 < T3 = T5 < T4. The outer metatarsal tubercle was absent, whereas the inner metatarsal tubercle was distinct and elongated.

We described the colouration from the live individual immediately after capture using field photographs. The dorsum was olive-brown to slaty brown. A conspicuous dark brown canthal stripe extended from the tip of the snout through the loreal region, across the eye, and into the tympanic region. The upper lip was creamish white with a pale pink tinge. The iris was golden pink with brown reticulations. The prominent dorsolateral folds were light slaty brown to pink on either side and narrowly bordered by dark brown. The flanks were dark olive-green to brownish, with numerous yellowish-grey speckles. The inguinal region is distinctly yellowish, contrasting with the darker flanks. Posterior surface of the thighs is dark brown with irregular pale-yellow mottling. Forelimbs and hind limbs are olive-brown with mixed dark transverse crossbars. Digits are light brown with faint, darker barring. Ventral surfaces creamy white to pale yellow; throat and anterior chest with very mild brown mottling; abdomen immaculate; inguinal region and proximal surfaces of the thighs distinctly yellow.

### 3.3. DNA Barcoding and Phylogenetic Analyses

**Figure 2:**
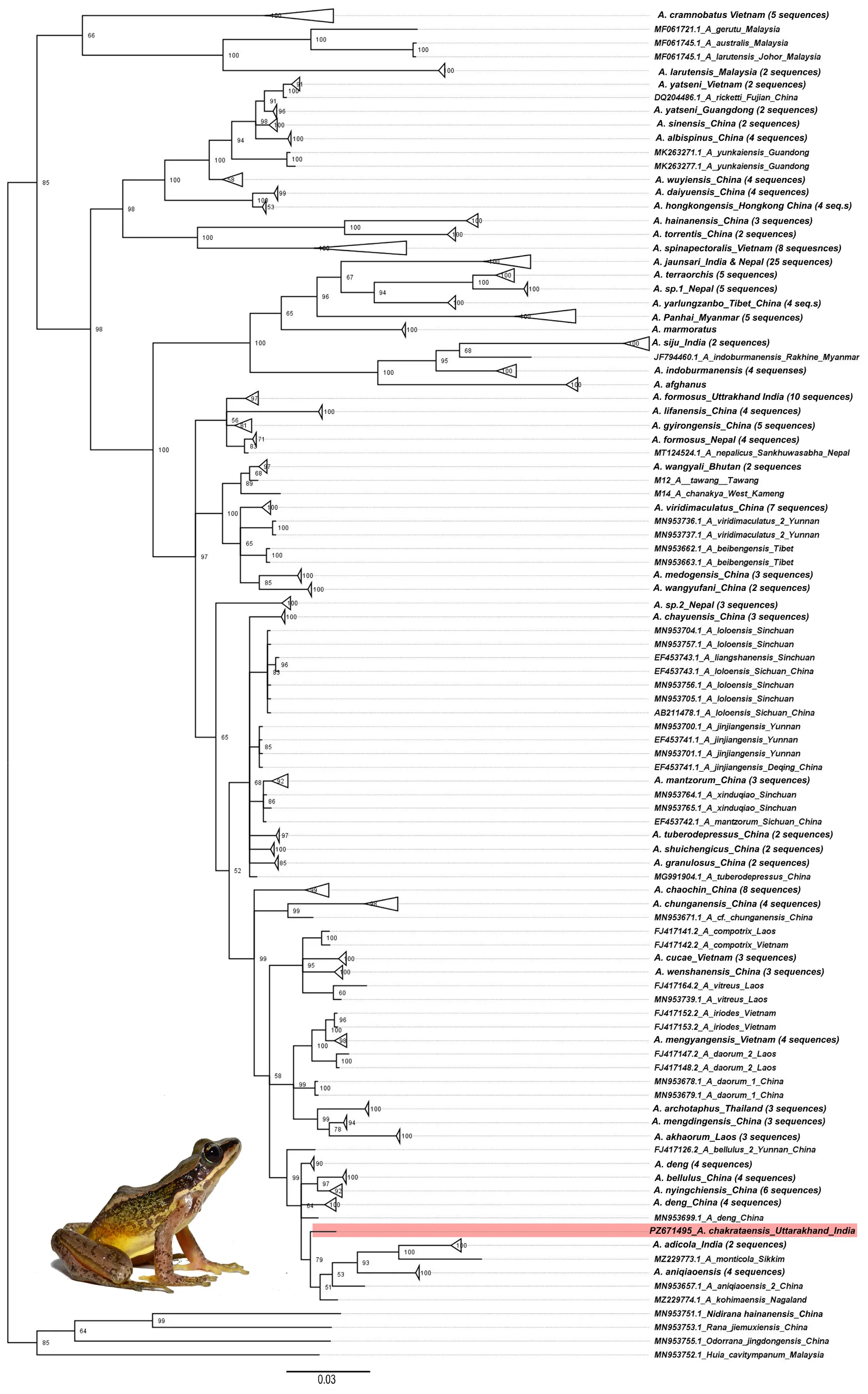
Bayesian phylogeny of *Amolops* based on a fragment of 433 bp of the mitochondrial 16S rRNA gene. Sequences generated in this study are highlighted (red) and labelled. GenBank accession numbers are shown for previously published sequences (Supplementary Table S2). Posterior probabilities are shown at nodes. Scale bar = substitutions per site.

The Bayesian phylogenetic analysis of the mitochondrial 16S rRNA gene fragment recovered *A. chakrataensis* as a distinct clade, accession number PZ671495 (Figure 2). The closely related lineages to *A. chakrataensis* were *A. kohimaensis*, *A. nyingchiensis*, *A. bellulus*, *A. deng*, and *A. aniqiaoensis*. Pairwise genetic distance analyses were congruent with the phylogenetic reconstruction (Table 2). The lowest uncorrected p-distance was observed between *A. chakrataensis* and *A. kohimaensis* (1.6%), followed by *A. nyingchiensis* (2.3%), *A. bellulus* (2.5%), and *A. deng* (2.8%). Mitochondrial genetic divergence from *A. aniqiaoensis*, *A*. cf. *formosus* (Uttarakhand), *A.* cf. *formosus* (Nepal), and *A. monticola* ranged from 4.2% to 5.9%, whereas the highest divergence among the selected taxa was observed with the sympatric species *A. jaunsari* (12.44%).

### 3.4. Potential Habitat Approximation

**Figure 3:**
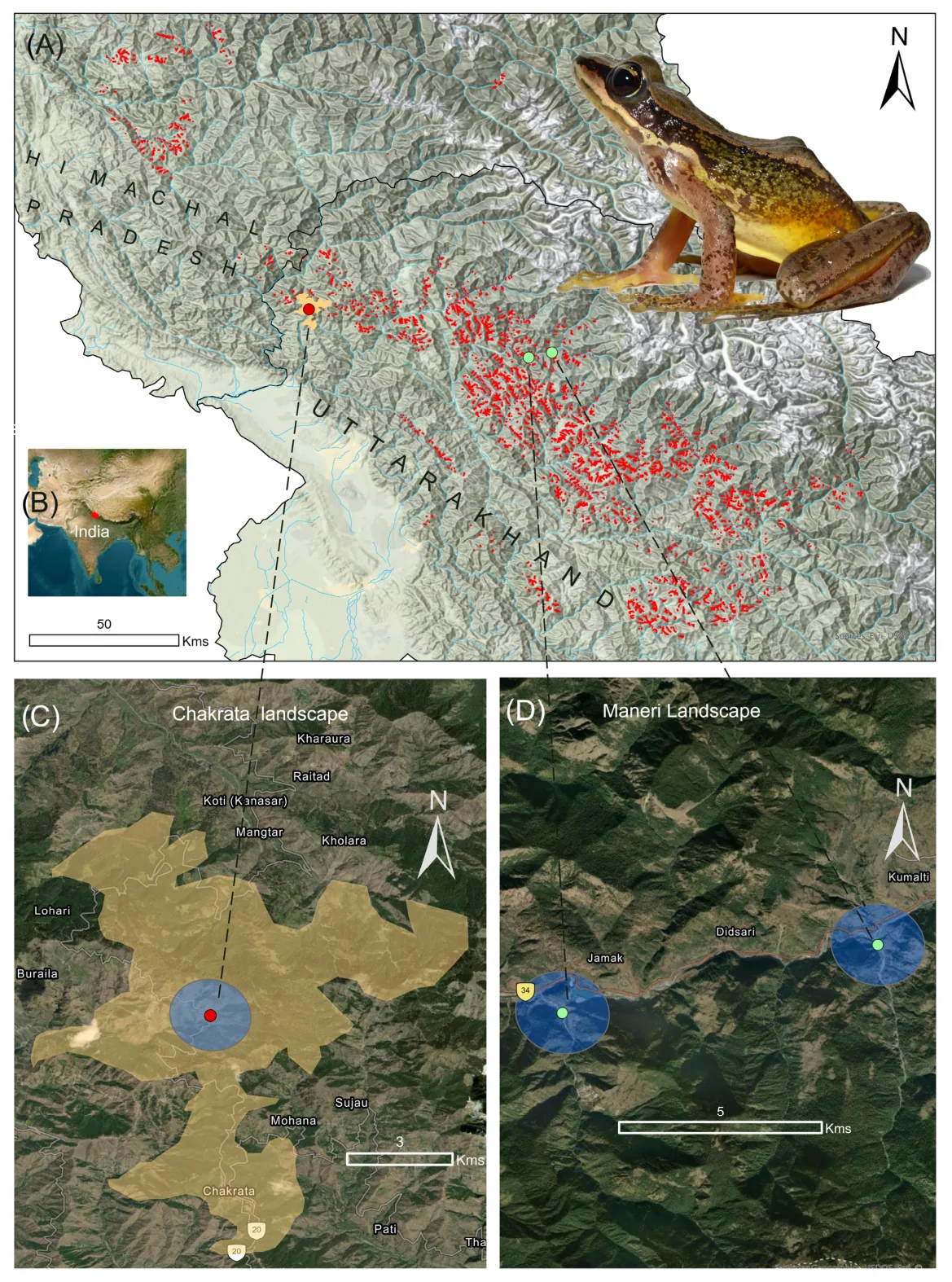
Known localities and predicted priority survey areas for *Amolops chakrataensis* in Uttarakhand and Himachal Pradesh, western Himalayas, India. (**A**) Map showing the type locality at Chakrata (red circle), the potential range identified from previous surveys (orange polygon), where *A. chakrataensis* was not detected despite repeated surveys [11], and the newly discovered localities in Maneri landscape, Uttarkashi District (green circles). Red patches indicate predicted priority areas for future surveys and conservation, identified using a habitat-envelope model based on the environmental characteristics of the three known localities. (**B**) Location of the study area within India. (**C**) Surveyed streams surrounding the type locality in the Chakrata landscape, Dehradun district (red circle). (**D**) Newly discovered localities in the Maneri landscape, Uttarkashi district (green circles). Blue shaded circles represent 1 km^2^ buffers around each known locality.

Our habitat-envelope-based approach identified several priority areas for future surveys and tentative conservation of *A. chakrataensis*, concentrated primarily around the new confirmed locality, together with adjacent stream sections where the additional individual was briefly observed (Figure 3). Based on the environmental characteristics of the three known localities, *A. chakrataensis* was associated with elevations ranging from 1320 to 2235 m asl, mean slope values of 16–24° (300 m resolution), flow accumulation values of 340–14,314, and forest cover of ≥42% within a 1 km buffer. The dominant lithological substrate across all three known localities was metamorphic rock [GLiM code: mt, 36].

## 4. Discussion

We rediscovered *Amolops chakrataensis* 41 years after its last confirmed record from the type locality in Chakrata [9,12]. The discovery of a new population in the Maneri landscape, approximately 65 km east of the type locality, extends the known distribution of *A. chakrataensis*. In addition, we acknowledge the possible sighting of the species by other non-focal research that provided an ambiguous picture of the species without confirming its identification [36]. Our rediscovery highlights the importance of continued field surveys in underexplored Himalayan landscapes [11]. In addition to the integrative taxonomic approach, we provide the first mitochondrial 16S rRNA sequence for *A. chakrataensis*, establishing an important genetic reference for future systematic studies.

The combination of morphology and DNA barcoding also provides an objective framework for distinguishing *A. chakrataensis* from morphologically similar congeners occurring in the western Himalayas. Importantly, we based the morphological identification on a combination of diagnostic morphological and meristic characters described in the original species description and observed through our direct examination of the holotype, rather than on individual morphometric measurements alone. We also compared the rediscovered individual with sympatric *Amolops* species and clearly distinguished it morphologically from these congeners based on diagnostic morphological and meristic characters, as well as the coloration (Figure 1; Table 1). We could not directly compare the holotype’s genetics as we did not have permission to obtain tissue samples. However, we recommend whole-genome sequencing to further resolve evolutionary relationships, as the 16S rRNA marker alone provides limited phylogenetic resolution for robust taxonomic inference [38].

In our phylogenetic analysis, although *A. kohimaensis* was recovered as the closest relative of *A. chakrataensis* based on the mitochondrial 16S dataset, both species are allopatric, with an aerial (straight-line) distance of more than approximately 1600 km separating their known distributions. The two species differ in several diagnostic morphological characters. *A. chakrataensis* is distinguished by its head being slightly wider than long (whereas head longer than wide in the case of *A. kohimaensis*), a relatively larger tympanum (approximately three-fifths of eye diameter; whereas a smaller tympanum in *A. kohimaensis*), absence of ventral microgranular patches (whereas extensive ventral microgranular patches in *A. kohimaensis*), and different dorsal and ventral colouration in *A. kohimaensis* [39]. These morphological differences further support the distinct taxonomic identity of *A. chakrataensis*.

Our habitat envelope analysis provides the first approximation of the environmental conditions associated with *A. chakrataensis*. All known localities occurred in forested headwater streams under metamorphic geology, with moderate slopes, relatively high forest cover, and similar hydrological characteristics (Figure 3). These environmental attributes identified several nearby areas with comparable habitat that represent priority locations for future surveys [33]. Although based on only three known localities, this habitat envelope offers a practical framework for directing future field efforts and informing conservation planning until additional occurrence records become available.

Despite extensive surveys covering 14 sites and 424 person-hours of effort across a broad elevational range, only a single individual was captured, whereas one additional individual was briefly observed. These findings suggest that *A. chakrataensis* is either highly localised or difficult to detect because of specialised habitat requirements or seasonal activity patterns. Although additional surveys across multiple seasons are urgently required to determine its population size and distribution, the extremely low detection rate highlights the need for continued monitoring and targeted conservation efforts. We recommend eDNA sampling for *A. chakrataensis* given its frequently higher detection capabilities [40,41].

Owing to the rarity and high conservation importance of the species, we did not collect the individual as a voucher specimen. Instead, we followed non-invasive sampling decisions aligned with best practices for high-risk species [21,42,43,44]. We documented the live individual through detailed photographs, morphometric measurements, and a non-invasive buccal swab for genetic analyses before releasing it at the site of capture. To minimise the risk of collection, we intentionally withheld the precise geographic coordinates of the rediscovered locality from this publication (Figure 3). The locality information will, however, be shared with the Uttarakhand Forest Department through the project report to facilitate future monitoring and conservation planning.

The rediscovery of *A. chakrataensis* further highlights the vulnerability of narrowly distributed Himalayan stream amphibians that depend on specialised freshwater habitats. Previous assessments have identified dams and water management and use, pollution, infrastructural development, and potential risks from invasive species and diseases as important threats to the species [10,11]. We have limited biological information on the ecology and behaviour of this rare, range-restricted anuran; targeted field surveys, habitat protection, and long-term monitoring are urgently required to identify remaining populations of *A. chakrataensis*.

The western Himalayas region is highly vulnerable to climate change, with glacial retreat, biodiversity loss, and altered hydrological regimes driving rapid environmental change [45,46]. Given the rarity and restricted distribution of *A. chakrataensis*, we strongly recommend continued surveys across the known localities and other environmentally suitable areas to identify additional populations, better understand the species’ distribution and population status, and refine its conservation requirements. These efforts should support the development of a local Conservation Action Plan for this threatened species and guide targeted conservation strategies in the western Himalayas through collaboration among scientists, government agencies, local communities, and other key stakeholders [47,48,49].

## 5. Conclusions

The rediscovery of *Amolops chakrataensis* after four decades was made possible through intensive, long-term field surveys conducted both within and beyond its previously known distribution. This finding extends the known distribution of the species beyond the type locality. By integrating morphology, mitochondrial DNA barcoding, and habitat-envelope analysis, this study provides the first comprehensive baseline for the species, including its first genetic reference sequence and an initial assessment of its environmental associations. Although *A. chakrataensis* remains extremely rare and was detected at only a single location in the Maneri landscape in Uttarakhand despite more than four years of extensive surveys across Uttarakhand in the western Himalayas, our findings provide an important foundation for future targeted surveys, ecological research, and conservation planning. Continued monitoring through field surveys, eDNA-based detection, and non-invasive sampling practices will be crucial for assessing the status of *A. chakrataensis*. Given its rarity and highly restricted distribution, we recommend establishing micro-reserves and recognising suitable areas as Other Effective Area-Based Conservation Measures (OECMs) for *A. chakrataensis* at its occurrence sites to provide targeted protection (49). These efforts, together with collaborative conservation actions involving researchers, government agencies, and local stakeholders, will be crucial for preventing the extinction of *A. chakrataensis* in the high-mountain ecosystems of the western Himalayas.

## Figures and Tables

**Table 1:** Morphometric measurements (mm) of the newly recorded individual of *Amolops chakrataensis* (sex unknown) compared with the holotype (NRS A-16, Zoological Survey of India, Northern Regional Centre, Dehradun; female). Measurements of the holotype are based on our re-examination of the specimen. Note: Measurements of the holotype are based on our re-examination of the specimen and may differ slightly from those reported in 1992 [9,12], such differences likely reflect long-term shrinkage of the preserved specimen in alcohol and minor inter-observer measurement variation.

Morphometric Characters	New Individual (Sex Unknown; mm)	Holotype (NRS A-16, ZSI–NRC Dehradun; Female; mm)
Snout–vent length (SVL)	58.1	53.2
Head length (HL)	17.9	18.4
Head width (HW)	19.1	18.9
Eye diameter (ED)	5.1	5.4
Tympanum diameter (TD)	3	2.8
Forelimb length (FLL)	13.1	11.7
Hand length (HAL)	17.6	15.4
Tibia length (TL)	35.1	33.0
Femur length (FL)	30.8	24.8
Foot length (FOL)	37.2	27.6
Inner metatarsal tubercle length (IMT)	2.9	2.6
Snout length (SL)	8.9	-
Internarial distance (IND)	7.6	-
Interorbital distance (IOD)	5.4	-

**Table 2:** Uncorrected pairwise genetic distances (p-distances; %) calculated from the mitochondrial 16S rRNA gene fragment between the single newly generated *Amolops chakrataensis* sequence and selected closely related *Amolops* species included in the phylogenetic analysis. Distances were calculated in MEGA X using the p-distance method. Values below the diagonal indicate percentage sequence divergence.

S. No.	Species	1	2	3	4	5	6	7	8	9	10	11	12
1	*A. chakrataensis*	0											
2	*A. formosus* (Uttarakhand)	4.7	0.0										
3	*A. jaunsari* (Uttarakhand)	12.4	12.4	0.0									
4	*A. jaunsari* (Uttarakhand)	12.4	12.4	0.0	0.0								
5	*A. adicola*	4.7	8	14.8	14.8	0.0							
6	*A. deng*	2.8	5.2	12.7	12.7	5.4	0.0						
7	*A. nyingchiensis*	2.3	5.2	12.9	12.9	5.6	1.8	0.0					
8	*A. bellulus*	2.6	5.6	13.4	13.4	5.4	2.6	1.2	0.0				
9	*A. aniqiaoensis*	4.2	5.4	12.9	131	4.7	4	4.2	4.9	0.0			
10	*A. monticola*	5.6	8.2	14.1	14.1	4	6.5	6.3	7	5.9	0.0		
11	*A. kohimaensis*	1.6	4.9	12.7	12.7	4.9	2.3	2.1	2.8	3.5	5.2	0.0	
12	*A. formosus* (Nepal)	4.9	1.9	12.4	12.4	7.7	5.4	5.4	5.4	5.9	8.4	5.2	0.0

## Data Availability

All data supporting the findings of this study are provided within the article and the accompanying Supplementary Materials. Additional information can be requested from the corresponding authors.

## Supplementary Materials

The following supporting information can be downloaded at: https://www.mdpi.com/article/10.3390/ani16193072/s1, Table S1. Accession numbers, species names, and reference of sequences used in this study. The table includes newly generated sequence (submitted to GenBank; accession numbers pending) together with reference sequences used for the phylogenetic analysis. Table S2. Details of sampling sites surveyed for Amolops chakrataensis in the Chakrata landscape (Dehradun district) and in the Bhagirathi River basin (Uttarkashi district), Uttarakhand, India, between 2023 and 2026. The table summarises geographic location, elevation, survey effort for A. chakrataensis, fieldwork dates and seasonal coverage, habitat characteristics, observed threats, and other amphibian species found at each site.
