# Supplementary material for: Rediscovery of the Lost and Critically Endangered Dehradun Stream Frog, Amolops chakrataensis
Source: Animals (Basel). 2026 Sep 29;16(19):3072. doi: 10.3390/ani16193072 (PMC13663422; doi:10.3390/ani16193072)
Supplement: Supplementary file 1 [file animals-16-03072-s001.zip › animals-4510555-supplementary.pdf]

Rediscovery of the Lost and Critically Endangered Dehradun Stream Frog, *Amolops chakrataensis*

Vishal Kumar Prasad [1,2,3,*], Saurav Chaudhary [2], Sky T. Button [4], Sumbul Gill [4], Amit Badola [5], Devendra Singh [2], Sandeep Kumar Gupta [2], K. P. Dinesh [6] and Amaël Borzée [3,4,7,*]

[1] Laboratory of Animal Behaviour and Conservation, College of Ecology and Environment, Nanjing Forestry University, Nanjing 210037, China
[2] Wildlife Institute of India, Dehradun 248001, Uttarakhand, India; sauravchaudhary533@gmail.com (S.C.); devurawat.dr@gmail.com (D.S.); skg@wii.gov.in (S.K.G.)
[3] Amphibian Specialist Group, Species Survival Commission, International Union for the Conservation of Nature, 1196 Gland, Switzerland
[4] Laboratory of Animal Behaviour and Conservation, College of Life Sciences, Nanjing Forestry University, Nanjing 210037, China; sky.tc.button@gmail.com (S.T.B.); sumbulgil067@gmail.com (S.G.)
[5] Biodiversity Research and Conservation Foundation, Dehradun 248001, Uttarakhand, India; amitbadola99@gmail.com
[6] Zoological Survey of India, Western Regional Centre, Pune 411007, Maharashtra, India; kpdinesh.zsi2@gmail.com
[7] Department of Zoology and General Biology, Faculty of Life Sciences, Fergana State University, Fergana 150100, Uzbekistan
* Correspondence: vishalprasad.wildlife@gmail.com (V.K.P.); amaelborzee@gmail.com (A.B.)

Supplementary Table S1. Accession numbers, species names, and reference of sequences used in this study. The table includes newly generated sequence (submitted to GenBank; accession numbers pending) together with reference sequences used for the phylogenetic analysis.

S.No.	Sample ID	Species	16S mtDNA sequences (GenBank accession numbers)	Reference	Full references
1	ACHAK	*A. chakrataensis*	PZ671495	This study	
2	ON209573_A_deng	*Amolops deng*	ON209573	Yu et al. (submitted 2022)	Yu, Z. B., Hou, S. B., Chen, T. E., An, D., Zhao ,H. P., Liu, Q., Xiong, Y., Zhu, L., Wu, Y. H. & Che, J. (Unpublished). Two New Records of *Amolops* in Gaoligongshan Mountains from Yunnan Province.
3	ON209577_A_deng	*Amolops deng*	ON209577	Yu et al. (submitted 2022)	Yu, Z. B., Hou, S. B., Chen, T. E., An, D., Zhao ,H. P., Liu, Q., Xiong, Y., Zhu, L., Wu, Y. H. & Che, J. (Unpublished). Two New Records of *Amolops* in Gaoligongshan Mountains from Yunnan Province.
4	FJ417127.2_A_bellulus	*Amolops bellulus*	FJ417127.2	Stuart et al., 2010	Stuart, B. L., Bain, R. H., Phimmachak, S., & Spence, K. (2010). Phylogenetic systematics of the *Amolops monticola* group (Amphibia: Ranidae), with description of a new species from northwestern Laos. *Herpetologica*, *66*(1), 52-66.
5	ON209572.1_A_deng	*Amolops deng*	ON209572.1	Yu et al. (submitted 2022)	Yu, Z. B., Hou, S. B., Chen, T. E., An, D., Zhao ,H. P., Liu, Q., Xiong, Y., Zhu, L., Wu, Y. H. & Che, J. (Unpublished). Two New Records of *Amolops* in Gaoligongshan Mountains from Yunnan Province.

6	ON209573.1_A_deng	*Amolops deng*	ON209573.1	Yu et al. (submitted 2022)	Yu, Z. B., Hou, S. B., Chen, T. E., An, D., Zhao ,H. P., Liu, Q., Xiong, Y., Zhu, L., Wu, Y. H. & Che, J. (Unpublished). Two New Records of *Amolops* in Gaoligongshan Mountains from Yunnan Province.
7	25A_CHAK	*Amolops formosus*	Needs to apply accession no.	Study under review	
8	Man02	*Amolops jaunsari*	Needs to apply accession no.	Study under review	
9	Man05	*Amolops jaunsari*	Needs to apply accession no.	Study under review	
10	Man03	*Amolops formosus*	Needs to apply accession no.	Study under review	
11	Man06	*Amolops formosus*	Needs to apply accession no.	Study under review	
12	25A_moli	*Amolops formosus*	Needs to apply accession no.	Study under review	
13	30C_moli	*Amolops formosus*	Needs to apply accession no.	Study under review	
14	30D_moli	*Amolops formosus*	Needs to apply accession no.	Study under review	
15	MUN2_A	*Amolops formosus*	Needs to apply accession no.	Study under review	
16	MUN2_B	*Amolops formosus*	Needs to apply accession no.	Study under review	
17	MUN2_D	*Amolops formosus*	Needs to apply accession no.	Study under review	
18	A.J.F.RAN1	*Amolops jaunsari*	OR801400	Study under review	
19	A2V2	*Amolops jaunsari*	OR801401	Study under review	
20	AC1	*Amolops jaunsari*	OR801402	Study under review	
21	AF2SI	*Amolops jaunsari*	OR801403	Study under review	
22	AFORM_01	*Amolops formosus*	OR801413	Study under review	
23	AJ_CHAFI_MAC	*Amolops jaunsari*	OR801404	Study under review	
24	AS1SBS	*Amolops jaunsari*	OR801406	Study under review	
25	AS2SBS	*Amolops jaunsari*	OR801407	Study under review	
26	AMSS	*Amolops jaunsari*	OR801405	Study under review	
27	ASF1	*Amolops jaunsari*	OR801408	Study under review	

28	NALA_JF	*Amolops jaunsari*	OR801412	Study under review	
29	MALAJH	*Amolops jaunsari*	OR801411	Study under review	
30	SAHAJ1	*Amolops jaunsari*	OR801409	Study under review	
31	SAHAJ2	*Amolops jaunsari*	OR801410	Study under review	Uttarakhand Amphibian paper
32	ON462447.1	*Amolops wangyali*	ON462447.1	Mahony et al., 2022	Mahony, S., Nidup, T., Streicher, J. W., Teeling, E. C., & Kamei, R. G. (2022). A review of torrent frogs (*Amolops*: Ranidae) from Bhutan, the description of a new species, and reassessment of the taxonomic validity of some *A. viridimaculatus* group species aided by archival DNA sequences of century-old type specimens. *Herpetological Journal*, (3).
33	ON462446.1	*Amolops wangyali*	ON462446.1	Mahony et al., 2022	Mahony, S., Nidup, T., Streicher, J. W., Teeling, E. C., & Kamei, R. G. (2022). A review of torrent frogs (*Amolops*: Ranidae) from Bhutan, the description of a new species, and reassessment of the taxonomic validity of some *A. viridimaculatus* group species aided by archival DNA sequences of century-old type specimens. *Herpetological Journal*, (3).
34	D_414_SIJU	*Amolops siju*	OM174173.1	Saikia et. al, 2023	Saikia, B., Sinha, B., Shabnam, A., & Dinesh, K. P. (2023). Description of a new species of *Amolops* (Anura: Ranidae) from a cave ecosystem in Meghalaya, Northeast India. *Journal of Animal Diversity*, *5*(1), 36-54.
35	D_414_SIJU2	*Amolops siju*	OM174172.1	Saikia et. al, 2023	Saikia, B., Sinha, B., Shabnam, A., & Dinesh, K. P. (2023). Description of a new species of *Amolops* (Anura: Ranidae) from a cave ecosystem in Meghalaya, Northeast India. *Journal of Animal Diversity*, *5*(1), 36-54.
36	D_331_A_terraorchis	*Amolops terraorchis*	MW794281.1	Saikia et al., 2022	Saikia, B., Sinha, B., Laskar, M. A., Shabnam, A., & Dinesh, K. P. (2022). A new species of *Amolops* (Anura: Ranidae) representing the morphological' Marmoratus species group' from Sessa Orchid Sanctuary, Arunachal Pradesh, Northeast India. *Records of the Zoological Survey of India*, 303-322.
37	D_333_A_terraorchis	*Amolops terraorchis*	MW794281.1	Saikia et al., 2022	Saikia, B., Sinha, B., Laskar, M. A., Shabnam, A., & Dinesh, K. P. (2022). A new species of *Amolops* (Anura: Ranidae) representing the morphological' Marmoratus species group' from Sessa Orchid Sanctuary, Arunachal Pradesh, Northeast India. *Records of the Zoological Survey of India*, 303-322.
38	D_334_A_terraorchis	*Amolops terraorchis*	MW794281.1	Saikia et al., 2022	Saikia, B., Sinha, B., Laskar, M. A., Shabnam, A., & Dinesh, K. P. (2022). A new species of *Amolops* (Anura: Ranidae) representing the morphological' Marmoratus species group' from Sessa Orchid Sanctuary, Arunachal Pradesh, Northeast India. *Records of the Zoological Survey of India*, 303-322.
39	D_335_A_terraorchis	*Amolops terraorchis*	MW794281.1	Saikia et al., 2022	Saikia, B., Sinha, B., Laskar, M. A., Shabnam, A., & Dinesh, K. P. (2022). A new species of *Amolops* (Anura: Ranidae) representing the morphological' Marmoratus species group' from Sessa Orchid Sanctuary, Arunachal Pradesh, Northeast India. *Records of the Zoological Survey of India*, 303-322.
40	D_336_A_terraorchis	*Amolops terraorchis*	MW794282.1	Saikia et al., 2022	Saikia, B., Sinha, B., Laskar, M. A., Shabnam, A., & Dinesh, K. P. (2022). A new species of *Amolops* (Anura: Ranidae) representing the morphological' Marmoratus species group' from Sessa Orchid Sanctuary, Arunachal Pradesh, Northeast India. *Records of the Zoological Survey of India*, 303-322.

41	M12_A__tawang__Tawang	*Amolops viridimaculatus*	ON025581	Saikia et al., 2022	Saikia, B., Sinha, B., Laskar, M. A., Shabnam, A., & Dinesh, K. P. (2022). A new species of *Amolops* (Anura: Ranidae) representing the morphological' Marmoratus species group' from Sessa Orchid Sanctuary, Arunachal Pradesh, Northeast India. *Records of the Zoological Survey of India*, 303-322.
42	M14_A_chanakya_West_Kameng	*Amolops viridimaculatus*	ON025582.1	Saikia et al., 2022	Saikia, B., Laskar, M. A., Dinesh, K. P., Shabnam, A., & Sinha, B. (2022). Description of two new species of *Amolops* (Anura: Ranidae) from Arunachal Pradesh, Northeast India under the morphological 'Viridimaculatus species group'. *Records of the Zoological Survey of India*, 247-266.
43	M15_A_gerbillus	*Amolops gerbillus*	OK138592.1	Laskar et al. (submitted 2021)	Laskar, M. A., Debnath, M., Sinha, B. & Saikia, B. (Unpublished). Biotech Department, St. Anthony's College, Bomfyle Road, Shillong, Meghalaya 793001, India.
44	M34_A_adicola	*Amolops adicola*	OK138593.1	Laskar et al. (submitted 2021)	Laskar, M. A., Debnath, M., Sinha, B. & Saikia, B. (Unpublished). Biotech Department, St. Anthony's College, Bomfyle Road, Shillong, Meghalaya 793001, India.
45	M35_A_aniqiaoensis_Upper_Subansiri	*Amolops aniqiaoensis*	MT636754.1	Laskar et al. (submitted 2020)	Laskar, M. A., Debnath, M., Das, H., Kharkongor, I. J. & Saikia, B. (Unpublished). Taxonomic Studies on Amphibians of North East India.
46	M36_A_aniqiaoensis_Upper_Subansiri	*Amolops aniqiaoensis*	MT636754.1	Laskar et al. (submitted 2020)	Laskar, M. A., Debnath, M., Das, H., Kharkongor, I. J. & Saikia, B. (Unpublished). Taxonomic Studies on Amphibians of North East India.
47	M50_A_indoburmanensis_Murlen_NP_Mizoram	*Amolops indoburmanensis*	MN953693.1	Wu et al., 2020	Wu, Y. H., Yan, F., Stuart, B. L., Prendini, E., Suwannapoom, C., Dahn, H. A., ... & Che, J. (2020). A combined approach of mitochondrial DNA and anchored nuclear phylogenomics sheds light on unrecognized diversity, phylogeny, and historical biogeography of the torrent frogs, genus *Amolops* (Anura: Ranidae). *Molecular phylogenetics and Evolution*, *148*, 106789.
48	KU840605.1_A_chaochin_china	*Amolops chunganensis*	KU840605.1	Goutte et al., 2016	Goutte, S., Dubois, A., Howard, S. D., Marquez, R., Rowley, J. J., Dehling, J. M., ... & Legendre, F. (2016). Environmental constraints and call evolution in torrent-dwelling frogs. *Evolution*, *70*(4), 811-826.
49	AB211477.1_A_chaochin	*Amolops chunganensis*	AB211477.1	Matsui et al., 2006	Matsui, M., Shimada, T., Liu, W. Z., Maryati, M., Khonsue, W., & Orlov, N. (2006). Phylogenetic relationships of Oriental torrent frogs in the genus *Amolops* and its allies (Amphibia, Anura, Ranidae). *Molecular Phylogenetics and Evolution*, *38*(3), 659-666.
50	KX645666.1_A_chaochin	*Amolops chunganensis*	KX645666.1	Yuan et al., 2016	Yuan, S., Xia, Y., Zheng, Y., & Zeng, X. (2016). Next-generation sequencing of mixed genomic DNA allows efficient assembly of rearranged mitochondrial genomes in *Amolops chunganensis* and *Quasipaa boulengeri*. *PeerJ*, *4*, e2786.
51	MN953668.1_A_chaochin_China	*Amolops chunganensis*	MN953668.1	Wu et al., 2020	Wu, Y. H., Yan, F., Stuart, B. L., Prendini, E., Suwannapoom, C., Dahn, H. A., ... & Che, J. (2020). A combined approach of mitochondrial DNA and anchored nuclear phylogenomics sheds light on unrecognized diversity, phylogeny, and historical biogeography of the torrent frogs, genus *Amolops* (Anura: Ranidae). *Molecular phylogenetics and Evolution*, *148*, 106789.
52	MN953669.1_A_chaochin_China	*Amolops chunganensis*	MN953669.1	Wu et al., 2020	Wu, Y. H., Yan, F., Stuart, B. L., Prendini, E., Suwannapoom, C., Dahn, H. A., ... & Che, J. (2020). A combined approach of mitochondrial DNA and anchored nuclear phylogenomics sheds light on unrecognized diversity, phylogeny, and historical biogeography of the torrent frogs, genus *Amolops* (Anura: Ranidae). *Molecular phylogenetics and Evolution*, *148*, 106789.
53	MN953766.1_A_chunganensis_China	*Amolops chunganensis*	MN953766.1	Wu et al., 2020	Wu, Y. H., Yan, F., Stuart, B. L., Prendini, E., Suwannapoom, C., Dahn, H. A., ... & Che, J. (2020). A combined approach of mitochondrial DNA and anchored nuclear phylogenomics sheds light on unrecognized diversity, phylogeny, and historical

					biogeography of the torrent frogs, genus *Amolops* (Anura: Ranidae). *Molecular phylogenetics and Evolution*, *148*, 106789.
54	KX645666.1_A_chaochin_China	*Amolops chunganensis*	KX645666.1	Yuan et al., 2016	Yuan, S., Xia, Y., Zheng, Y., & Zeng, X. (2016). Next-generation sequencing of mixed genomic DNA allows efficient assembly of rearranged mitochondrial genomes in *Amolops chunganensis* and *Quasipaa boulengeri*. *PeerJ*, *4*, e2786.
55	MN953767.1_A_chaochin_China	*Amolops chunganensis*	MN953767.1	Wu et al., 2020	Wu, Y. H., Yan, F., Stuart, B. L., Prendini, E., Suwannapoom, C., Dahn, H. A., ... & Che, J. (2020). A combined approach of mitochondrial DNA and anchored nuclear phylogenomics sheds light on unrecognized diversity, phylogeny, and historical biogeography of the torrent frogs, genus *Amolops* (Anura: Ranidae). *Molecular phylogenetics and Evolution*, *148*, 106789.
56	MG991886.1_A_chunganensis_China	*Amolops chunganensis*	MG991886.1	Lyu et al., 2018	Lyu, Z. T., Wu, J., Wang, J., Sung, Y. H., Liu, Z. Y., Zeng, Z. C., ... & Wang, Y. Y. (2018). A new species of *Amolops* (Anura: Ranidae) from southwestern Guangdong, China. *Zootaxa*, *4418*(6), 562-576.
57	KX507310.1_A_chunganensis_vChina	*Amolops chunganensis*	KX507310.1	Sung et al., 2016	Sung, Y. H., Hu, P., Wang, J., Liu, H. J., & Wang, Y. Y. (2016). A new species of *Amolops* (Anura: Ranidae) from southern China. *Zootaxa*, *4170*(3), 525-538.
58	MN953670.1_A_chunganensis_China	*Amolops chunganensis*	MN953670.1	Wu et al., 2020	Wu, Y. H., Yan, F., Stuart, B. L., Prendini, E., Suwannapoom, C., Dahn, H. A., ... & Che, J. (2020). A combined approach of mitochondrial DNA and anchored nuclear phylogenomics sheds light on unrecognized diversity, phylogeny, and historical biogeography of the torrent frogs, genus *Amolops* (Anura: Ranidae). *Molecular phylogenetics and Evolution*, *148*, 106789.
59	MN953762.1_A_chunganensis_China	*Amolops chunganensis*	MN953762.1	Wu et al., 2020	Wu, Y. H., Yan, F., Stuart, B. L., Prendini, E., Suwannapoom, C., Dahn, H. A., ... & Che, J. (2020). A combined approach of mitochondrial DNA and anchored nuclear phylogenomics sheds light on unrecognized diversity, phylogeny, and historical biogeography of the torrent frogs, genus *Amolops* (Anura: Ranidae). *Molecular phylogenetics and Evolution*, *148*, 106789.
60	MN953671.1_A_cf._chunganensis_China	*Amolops chunganensis*	MN953671.1	Wu et al., 2020	Wu, Y. H., Yan, F., Stuart, B. L., Prendini, E., Suwannapoom, C., Dahn, H. A., ... & Che, J. (2020). A combined approach of mitochondrial DNA and anchored nuclear phylogenomics sheds light on unrecognized diversity, phylogeny, and historical biogeography of the torrent frogs, genus *Amolops* (Anura: Ranidae). *Molecular phylogenetics and Evolution*, *148*, 106789.
61	FJ417141.2_A_compotrix_Laos	*Amolops compotrix*	FJ417141.2	Stuart et al., 2010	Stuart, B. L., Bain, R. H., Phimmachak, S., & Spence, K. (2010). Phylogenetic systematics of the *Amolops monticola* group (Amphibia: Ranidae), with description of a new species from northwestern Laos. *Herpetologica*, *66*(1), 52-66.
62	FJ417142.2_A_compotrix_Vietnam	*Amolops compotrix*	FJ417142.2	Stuart et al., 2010	Stuart, B. L., Bain, R. H., Phimmachak, S., & Spence, K. (2010). Phylogenetic systematics of the *Amolops monticola* group (Amphibia: Ranidae), with description of a new species from northwestern Laos. *Herpetologica*, *66*(1), 52-66.
63	FJ417145.2_A_cucae_Vietnam	*Amolops cucae*	FJ417145.2	Stuart et al., 2010	Stuart, B. L., Bain, R. H., Phimmachak, S., & Spence, K. (2010). Phylogenetic systematics of the *Amolops monticola* group (Amphibia: Ranidae), with description of a new species from northwestern Laos. *Herpetologica*, *66*(1), 52-66.
64	FJ417144.2_A_cucae_Vietnam	*Amolops cucae*	FJ417144.2	Stuart et al., 2010	Stuart, B. L., Bain, R. H., Phimmachak, S., & Spence, K. (2010). Phylogenetic systematics of the *Amolops monticola* group (Amphibia: Ranidae), with description of a new species from northwestern Laos. *Herpetologica*, *66*(1), 52-66.
65	MN953724.1_A_wenshanensis_China	*Amolops wenshanensis*	MN953724.1	Wu et al., 2020	Wu, Y. H., Yan, F., Stuart, B. L., Prendini, E., Suwannapoom, C., Dahn, H. A., ... & Che, J. (2020). A combined approach of mitochondrial DNA and anchored nuclear phylogenomics sheds light on unrecognized diversity, phylogeny, and historical

					biogeography of the torrent frogs, genus *Amolops* (Anura: Ranidae). *Molecular phylogenetics and Evolution*, *148*, 106789.
66	MN953725.1_A_wenshanensis_China	*Amolops wenshanensis*	MN953725.1	Wu et al., 2020	Wu, Y. H., Yan, F., Stuart, B. L., Prendini, E., Suwannapoom, C., Dahn, H. A., ... & Che, J. (2020). A combined approach of mitochondrial DNA and anchored nuclear phylogenomics sheds light on unrecognized diversity, phylogeny, and historical biogeography of the torrent frogs, genus *Amolops* (Anura: Ranidae). *Molecular phylogenetics and Evolution*, *148*, 106789.
67	FJ417129.2_A_wenshanensis_China	*Amolops wenshanensis*	FJ417129.2	Stuart et al., 2010	Stuart, B. L., Bain, R. H., Phimmachak, S., & Spence, K. (2010). Phylogenetic systematics of the *Amolops monticola* group (Amphibia: Ranidae), with description of a new species from northwestern Laos. *Herpetologica*, *66*(1), 52-66.
68	FJ417164.2_A_vitreus_Laos	*Amolops vitreus*	FJ417164.2	Stuart et al., 2010	Stuart, B. L., Bain, R. H., Phimmachak, S., & Spence, K. (2010). Phylogenetic systematics of the *Amolops monticola* group (Amphibia: Ranidae), with description of a new species from northwestern Laos. *Herpetologica*, *66*(1), 52-66.
69	MN953739.1_A_vitreus_Laos	*Amolops vitreus*	MN953739.1	Wu et al., 2020	Wu, Y. H., Yan, F., Stuart, B. L., Prendini, E., Suwannapoom, C., Dahn, H. A., ... & Che, J. (2020). A combined approach of mitochondrial DNA and anchored nuclear phylogenomics sheds light on unrecognized diversity, phylogeny, and historical biogeography of the torrent frogs, genus *Amolops* (Anura: Ranidae). *Molecular phylogenetics and Evolution*, *148*, 106789.
70	FJ417152.2_A_iriodes_Vietnam	*Amolops iriodes*	FJ417152.2	Stuart et al., 2010	Stuart, B. L., Bain, R. H., Phimmachak, S., & Spence, K. (2010). Phylogenetic systematics of the *Amolops monticola* group (Amphibia: Ranidae), with description of a new species from northwestern Laos. *Herpetologica*, *66*(1), 52-66.
71	FJ417153.2_A_iriodes_Vietnam	*Amolops iriodes*	FJ417153.2	Stuart et al., 2010	Stuart, B. L., Bain, R. H., Phimmachak, S., & Spence, K. (2010). Phylogenetic systematics of the *Amolops monticola* group (Amphibia: Ranidae), with description of a new species from northwestern Laos. *Herpetologica*, *66*(1), 52-66.
72	KR827703.1_A_mengyangensis_Vietnam	*Amolops mengyangensis*	KR827703.1	Grosjean et al., 2015	Grosjean, S., Ohler, A., Chuaynkern, Y., Cruaud, C., & Hassanin, A. (2015). Improving biodiversity assessment of anuran amphibians using DNA barcoding of tadpoles. Case studies from Southeast Asia. *Comptes Rendus. Biologies*, *338*(5), 351-361.
73	KR827704.1_A_mengyangensis_Vietnam	*Amolops mengyangensis*	KR827704.1	Grosjean et al., 2015	Grosjean, S., Ohler, A., Chuaynkern, Y., Cruaud, C., & Hassanin, A. (2015). Improving biodiversity assessment of anuran amphibians using DNA barcoding of tadpoles. Case studies from Southeast Asia. *Comptes Rendus. Biologies*, *338*(5), 351-361.
74	MN953678.1_A_daorum_1_China	*Amolops daorum*	MN953678.1	Wu et al., 2020	Wu, Y. H., Yan, F., Stuart, B. L., Prendini, E., Suwannapoom, C., Dahn, H. A., ... & Che, J. (2020). A combined approach of mitochondrial DNA and anchored nuclear phylogenomics sheds light on unrecognized diversity, phylogeny, and historical biogeography of the torrent frogs, genus *Amolops* (Anura: Ranidae). *Molecular phylogenetics and Evolution*, *148*, 106789.
75	MN953679.1_A_daorum_1_China	*Amolops daorum*	MN953679.1	Wu et al., 2020	Wu, Y. H., Yan, F., Stuart, B. L., Prendini, E., Suwannapoom, C., Dahn, H. A., ... & Che, J. (2020). A combined approach of mitochondrial DNA and anchored nuclear phylogenomics sheds light on unrecognized diversity, phylogeny, and historical biogeography of the torrent frogs, genus *Amolops* (Anura: Ranidae). *Molecular phylogenetics and Evolution*, *148*, 106789.
76	FJ417151.2_A_mengyangensis_Vietnam	*Amolops daorum*	FJ417151.2	Stuart et al., 2010	Stuart, B. L., Bain, R. H., Phimmachak, S., & Spence, K. (2010). Phylogenetic systematics of the *Amolops monticola* group (Amphibia: Ranidae), with description of a new species from northwestern Laos. *Herpetologica*, *66*(1), 52-66.

77	FJ417147.2_A_daorum_2_Laos	*Amolops daorum*	FJ417147.2	Stuart et al., 2010	Stuart, B. L., Bain, R. H., Phimmachak, S., & Spence, K. (2010). Phylogenetic systematics of the *Amolops monticola* group (Amphibia: Ranidae), with description of a new species from northwestern Laos. *Herpetologica*, *66*(1), 52-66.
78	FJ417148.2_A_daorum_2_Laos	*Amolops daorum*	FJ417148.2	Stuart et al., 2010	Stuart, B. L., Bain, R. H., Phimmachak, S., & Spence, K. (2010). Phylogenetic systematics of the *Amolops monticola* group (Amphibia: Ranidae), with description of a new species from northwestern Laos. *Herpetologica*, *66*(1), 52-66.
79	MN953659.1_A_archotaphus_Thailand	*Amolops archotaphus*	MN953659.1	Wu et al., 2020	Wu, Y. H., Yan, F., Stuart, B. L., Prendini, E., Suwannapoom, C., Dahn, H. A., ... & Che, J. (2020). A combined approach of mitochondrial DNA and anchored nuclear phylogenomics sheds light on unrecognized diversity, phylogeny, and historical biogeography of the torrent frogs, genus *Amolops* (Anura: Ranidae). *Molecular phylogenetics and Evolution*, *148*, 106789.
80	MN953660.1_A_archotaphus_Thailand	*Amolops archotaphus*	MN953660.1	Wu et al., 2020	Wu, Y. H., Yan, F., Stuart, B. L., Prendini, E., Suwannapoom, C., Dahn, H. A., ... & Che, J. (2020). A combined approach of mitochondrial DNA and anchored nuclear phylogenomics sheds light on unrecognized diversity, phylogeny, and historical biogeography of the torrent frogs, genus *Amolops* (Anura: Ranidae). *Molecular phylogenetics and Evolution*, *148*, 106789.
81	MN953661.1_A_archotaphus_Thailand	*Amolops archotaphus*	MN953661.1	Wu et al., 2020	Wu, Y. H., Yan, F., Stuart, B. L., Prendini, E., Suwannapoom, C., Dahn, H. A., ... & Che, J. (2020). A combined approach of mitochondrial DNA and anchored nuclear phylogenomics sheds light on unrecognized diversity, phylogeny, and historical biogeography of the torrent frogs, genus *Amolops* (Anura: Ranidae). *Molecular phylogenetics and Evolution*, *148*, 106789.
82	MK501809.1_A_mengdingensis_China	*Amolops mengdingensis*	MK501809.1	Yu et al., 2019	Yu, G., Wu, Z., & Yang, J. (2019). A new species of the *Amolops monticola* group (Anura: Ranidae) from southwestern Yunnan, China. *Zootaxa*, *4577*(3), zootaxa-4577.
83	MK501810.1_A_mengdingensis_China	*Amolops mengdingensis*	MK501810.1	Yu et al., 2019	Yu, G., Wu, Z., & Yang, J. (2019). A new species of the *Amolops monticola* group (Anura: Ranidae) from southwestern Yunnan, China. *Zootaxa*, *4577*(3), zootaxa-4577.
84	MK501808.1_A_mengdingensis_China	*Amolops mengdingensis*	MK501808.1	Yu et al., 2019	Yu, G., Wu, Z., & Yang, J. (2019). A new species of the *Amolops monticola* group (Anura: Ranidae) from southwestern Yunnan, China. *Zootaxa*, *4577*(3), zootaxa-4577.
85	FJ417158.2_A_akhaorum_Laos	*Amolops akhaorum*	FJ417158.2	Stuart et al., 2010	Stuart, B. L., Bain, R. H., Phimmachak, S., & Spence, K. (2010). Phylogenetic systematics of the *Amolops monticola* group (Amphibia: Ranidae), with description of a new species from northwestern Laos. *Herpetologica*, *66*(1), 52-66.
86	FJ417159.2_A_akhaorum_Laos	*Amolops akhaorum*	FJ417159.2	Stuart et al., 2010	Stuart, B. L., Bain, R. H., Phimmachak, S., & Spence, K. (2010). Phylogenetic systematics of the *Amolops monticola* group (Amphibia: Ranidae), with description of a new species from northwestern Laos. *Herpetologica*, *66*(1), 52-66.
87	MN953695.1_A_deng_China	*Amolops sp. 1 YW-2020*	MN953695.1	Wu et al., 2020	Wu, Y. H., Yan, F., Stuart, B. L., Prendini, E., Suwannapoom, C., Dahn, H. A., ... & Che, J. (2020). A combined approach of mitochondrial DNA and anchored nuclear phylogenomics sheds light on unrecognized diversity, phylogeny, and historical biogeography of the torrent frogs, genus *Amolops* (Anura: Ranidae). *Molecular phylogenetics and Evolution*, *148*, 106789.
88	MN953696.1_A_deng_China	*Amolops sp. 1 YW-2020*	MN953696.1	Wu et al., 2020	Wu, Y. H., Yan, F., Stuart, B. L., Prendini, E., Suwannapoom, C., Dahn, H. A., ... & Che, J. (2020). A combined approach of mitochondrial DNA and anchored nuclear phylogenomics sheds light on unrecognized diversity, phylogeny, and historical biogeography of the torrent frogs, genus *Amolops* (Anura: Ranidae). *Molecular phylogenetics and Evolution*, *148*, 106789.
89	MN953697.1_A_deng_China	*Amolops sp. 1 YW-2020*	MN953697.1	Wu et al., 2020	Wu, Y. H., Yan, F., Stuart, B. L., Prendini, E., Suwannapoom, C., Dahn, H. A., ... & Che, J. (2020). A combined approach of mitochondrial DNA and anchored nuclear

					phylogenomics sheds light on unrecognized diversity, phylogeny, and historical biogeography of the torrent frogs, genus *Amolops* (Anura: Ranidae). *Molecular phylogenetics and Evolution*, *148*, 106789.
90	MN953698.1_A_deng_China	*Amolops sp. 1 YW-2020*	MN953698.1	Wu et al., 2020	Wu, Y. H., Yan, F., Stuart, B. L., Prendini, E., Suwannapoom, C., Dahn, H. A., ... & Che, J. (2020). A combined approach of mitochondrial DNA and anchored nuclear phylogenomics sheds light on unrecognized diversity, phylogeny, and historical biogeography of the torrent frogs, genus *Amolops* (Anura: Ranidae). *Molecular phylogenetics and Evolution*, *148*, 106789.
91	MN953699.1_A_deng_China	*Amolops sp. 1 YW-2020*	MN953699.1	Wu et al., 2020	Wu, Y. H., Yan, F., Stuart, B. L., Prendini, E., Suwannapoom, C., Dahn, H. A., ... & Che, J. (2020). A combined approach of mitochondrial DNA and anchored nuclear phylogenomics sheds light on unrecognized diversity, phylogeny, and historical biogeography of the torrent frogs, genus *Amolops* (Anura: Ranidae). *Molecular phylogenetics and Evolution*, *148*, 106789.
92	MN953716.1_A_nyingchiensis_China	*Amolops nyingchiensis*	MN953716.1	Wu et al., 2020	Wu, Y. H., Yan, F., Stuart, B. L., Prendini, E., Suwannapoom, C., Dahn, H. A., ... & Che, J. (2020). A combined approach of mitochondrial DNA and anchored nuclear phylogenomics sheds light on unrecognized diversity, phylogeny, and historical biogeography of the torrent frogs, genus *Amolops* (Anura: Ranidae). *Molecular phylogenetics and Evolution*, *148*, 106789.
93	MN953719.1_A_nyingchiensis_China	*Amolops nyingchiensis*	MN953719.1	Wu et al., 2020	Wu, Y. H., Yan, F., Stuart, B. L., Prendini, E., Suwannapoom, C., Dahn, H. A., ... & Che, J. (2020). A combined approach of mitochondrial DNA and anchored nuclear phylogenomics sheds light on unrecognized diversity, phylogeny, and historical biogeography of the torrent frogs, genus *Amolops* (Anura: Ranidae). *Molecular phylogenetics and Evolution*, *148*, 106789.
94	MN953715.1_A_nyingchiensis_China	*Amolops nyingchiensis*	MN953715.1	Wu et al., 2020	Wu, Y. H., Yan, F., Stuart, B. L., Prendini, E., Suwannapoom, C., Dahn, H. A., ... & Che, J. (2020). A combined approach of mitochondrial DNA and anchored nuclear phylogenomics sheds light on unrecognized diversity, phylogeny, and historical biogeography of the torrent frogs, genus *Amolops* (Anura: Ranidae). *Molecular phylogenetics and Evolution*, *148*, 106789.
95	MN953717.1_A_nyingchiensis_China	*Amolops nyingchiensis*	MN953717.1	Wu et al., 2020	Wu, Y. H., Yan, F., Stuart, B. L., Prendini, E., Suwannapoom, C., Dahn, H. A., ... & Che, J. (2020). A combined approach of mitochondrial DNA and anchored nuclear phylogenomics sheds light on unrecognized diversity, phylogeny, and historical biogeography of the torrent frogs, genus *Amolops* (Anura: Ranidae). *Molecular phylogenetics and Evolution*, *148*, 106789.
96	MN953718.1_A_nyingchiensis_China	*Amolops nyingchiensis*	MN953718.1	Wu et al., 2020	Wu, Y. H., Yan, F., Stuart, B. L., Prendini, E., Suwannapoom, C., Dahn, H. A., ... & Che, J. (2020). A combined approach of mitochondrial DNA and anchored nuclear phylogenomics sheds light on unrecognized diversity, phylogeny, and historical biogeography of the torrent frogs, genus *Amolops* (Anura: Ranidae). *Molecular phylogenetics and Evolution*, *148*, 106789.
97	MN953664.1_A_bellulus_1_China	*Amolops bellulus*	MN953664.1	Wu et al., 2020	Wu, Y. H., Yan, F., Stuart, B. L., Prendini, E., Suwannapoom, C., Dahn, H. A., ... & Che, J. (2020). A combined approach of mitochondrial DNA and anchored nuclear phylogenomics sheds light on unrecognized diversity, phylogeny, and historical biogeography of the torrent frogs, genus *Amolops* (Anura: Ranidae). *Molecular phylogenetics and Evolution*, *148*, 106789.
98	MN953665.1_A_bellulus_1_China	*Amolops bellulus*	MN953665.1	Wu et al., 2020	Wu, Y. H., Yan, F., Stuart, B. L., Prendini, E., Suwannapoom, C., Dahn, H. A., ... & Che, J. (2020). A combined approach of mitochondrial DNA and anchored nuclear

					phylogenomics sheds light on unrecognized diversity, phylogeny, and historical biogeography of the torrent frogs, genus *Amolops* (Anura: Ranidae). *Molecular phylogenetics and Evolution*, *148*, 106789.
99	FJ417127.2_A_bellulus_1_China	*Amolops bellulus*	FJ417127.2	Stuart et al., 2010	Stuart, B. L., Bain, R. H., Phimmachak, S., & Spence, K. (2010). Phylogenetic systematics of the *Amolops monticola* group (Amphibia: Ranidae), with description of a new species from northwestern Laos. *Herpetologica*, *66*(1), 52-66.
100	MN953655.1_A_aniqiaoensis_1_China	*Amolops aniqiaoensis*	MN953655.1	Wu et al., 2020	Wu, Y. H., Yan, F., Stuart, B. L., Prendini, E., Suwannapoom, C., Dahn, H. A., ... & Che, J. (2020). A combined approach of mitochondrial DNA and anchored nuclear phylogenomics sheds light on unrecognized diversity, phylogeny, and historical biogeography of the torrent frogs, genus *Amolops* (Anura: Ranidae). *Molecular phylogenetics and Evolution*, *148*, 106789.
101	MN953656.1_A_aniqiaoensis_1_China	*Amolops aniqiaoensis*	MN953656.1	Wu et al., 2020	Wu, Y. H., Yan, F., Stuart, B. L., Prendini, E., Suwannapoom, C., Dahn, H. A., ... & Che, J. (2020). A combined approach of mitochondrial DNA and anchored nuclear phylogenomics sheds light on unrecognized diversity, phylogeny, and historical biogeography of the torrent frogs, genus *Amolops* (Anura: Ranidae). *Molecular phylogenetics and Evolution*, *148*, 106789.
102	MN953658.1_A_aniqiaoensis_1_China	*Amolops aniqiaoensis*	MN953658.1	Wu et al., 2020	Wu, Y. H., Yan, F., Stuart, B. L., Prendini, E., Suwannapoom, C., Dahn, H. A., ... & Che, J. (2020). A combined approach of mitochondrial DNA and anchored nuclear phylogenomics sheds light on unrecognized diversity, phylogeny, and historical biogeography of the torrent frogs, genus *Amolops* (Anura: Ranidae). *Molecular phylogenetics and Evolution*, *148*, 106789.
103	MN953657.1_A_aniqiaoensis_2_China	*Amolops aniqiaoensis*	MN953657.1	Wu et al., 2020	Wu, Y. H., Yan, F., Stuart, B. L., Prendini, E., Suwannapoom, C., Dahn, H. A., ... & Che, J. (2020). A combined approach of mitochondrial DNA and anchored nuclear phylogenomics sheds light on unrecognized diversity, phylogeny, and historical biogeography of the torrent frogs, genus *Amolops* (Anura: Ranidae). *Molecular phylogenetics and Evolution*, *148*, 106789.
104	FJ417126.2_A_bellulus_2_Yunnan_China	*Amolops bellulus*	FJ417126.2	Stuart et al., 2010	Stuart, B. L., Bain, R. H., Phimmachak, S., & Spence, K. (2010). Phylogenetic systematics of the *Amolops monticola* group (Amphibia: Ranidae), with description of a new species from northwestern Laos. *Herpetologica*, *66*(1), 52-66.
105	MN953666.1_A_chayuensis_Tibet	*Amolops chayuensis*	MN953666.1	Wu et al., 2020	Wu, Y. H., Yan, F., Stuart, B. L., Prendini, E., Suwannapoom, C., Dahn, H. A., ... & Che, J. (2020). A combined approach of mitochondrial DNA and anchored nuclear phylogenomics sheds light on unrecognized diversity, phylogeny, and historical biogeography of the torrent frogs, genus *Amolops* (Anura: Ranidae). *Molecular phylogenetics and Evolution*, *148*, 106789.
106	MN953667.1_A_chayuensis_Tibet	*Amolops chayuensis*	MN953667.1	Wu et al., 2020	Wu, Y. H., Yan, F., Stuart, B. L., Prendini, E., Suwannapoom, C., Dahn, H. A., ... & Che, J. (2020). A combined approach of mitochondrial DNA and anchored nuclear phylogenomics sheds light on unrecognized diversity, phylogeny, and historical biogeography of the torrent frogs, genus *Amolops* (Anura: Ranidae). *Molecular phylogenetics and Evolution*, *148*, 106789.
107	MN953704.1_A_loloensis_Sinchuan	*Amolops loloensis*	MN953704.1	Wu et al., 2020	Wu, Y. H., Yan, F., Stuart, B. L., Prendini, E., Suwannapoom, C., Dahn, H. A., ... & Che, J. (2020). A combined approach of mitochondrial DNA and anchored nuclear phylogenomics sheds light on unrecognized diversity, phylogeny, and historical biogeography of the torrent frogs, genus *Amolops* (Anura: Ranidae). *Molecular phylogenetics and Evolution*, *148*, 106789.

108	MN953757.1_A_loloensis_Sinchuan	*Amolops loloensis*	MN953757.1	Wu et al., 2020	Wu, Y. H., Yan, F., Stuart, B. L., Prendini, E., Suwannapoom, C., Dahn, H. A., ... & Che, J. (2020). A combined approach of mitochondrial DNA and anchored nuclear phylogenomics sheds light on unrecognized diversity, phylogeny, and historical biogeography of the torrent frogs, genus *Amolops* (Anura: Ranidae). *Molecular phylogenetics and Evolution*, *148*, 106789.
109	EF453743.1_A_liangshanensis_Sinchuan	*Amolops liangshanensis*	EF453743.1	Cai & Zhang (submitted 2007)	Cai, H., & Zhang, Y. (Unpublished). A Re-analysis of the Phylogeny of the Genus *Amolops* (Anura, Ranidae), with Particular Reference to the Systematic Position of Wurana tormota.
110	MN953756.1_A_loloensis_Sinchuan	*Amolops loloensis*	MN953756.1	Wu et al., 2020	Wu, Y. H., Yan, F., Stuart, B. L., Prendini, E., Suwannapoom, C., Dahn, H. A., ... & Che, J. (2020). A combined approach of mitochondrial DNA and anchored nuclear phylogenomics sheds light on unrecognized diversity, phylogeny, and historical biogeography of the torrent frogs, genus *Amolops* (Anura: Ranidae). *Molecular phylogenetics and Evolution*, *148*, 106789.
111	MN953705.1_A_loloensis_Sinchuan	*Amolops loloensis*	MN953705.1	Wu et al., 2020	Wu, Y. H., Yan, F., Stuart, B. L., Prendini, E., Suwannapoom, C., Dahn, H. A., ... & Che, J. (2020). A combined approach of mitochondrial DNA and anchored nuclear phylogenomics sheds light on unrecognized diversity, phylogeny, and historical biogeography of the torrent frogs, genus *Amolops* (Anura: Ranidae). *Molecular phylogenetics and Evolution*, *148*, 106789.
112	MN953700.1_A_jinjiangensis_Yunnan	*Amolops jinjiangensis*	MN953700.1	Wu et al., 2020	Wu, Y. H., Yan, F., Stuart, B. L., Prendini, E., Suwannapoom, C., Dahn, H. A., ... & Che, J. (2020). A combined approach of mitochondrial DNA and anchored nuclear phylogenomics sheds light on unrecognized diversity, phylogeny, and historical biogeography of the torrent frogs, genus *Amolops* (Anura: Ranidae). *Molecular phylogenetics and Evolution*, *148*, 106789.
113	EF453741.1_A_jinjiangensis_Yunnan	*Amolops jinjiangensis*	EF453741.1	Cai & Zhang (submitted 2007)	Cai, H., & Zhang, Y. (Unpublished). A Re-analysis of the Phylogeny of the Genus *Amolops* (Anura, Ranidae), with Particular Reference to the Systematic Position of Wurana tormota.
114	MN953701.1_A_jinjiangensis_Yunnan	*Amolops jinjiangensis*	MN953701.1	Wu et al., 2020	Wu, Y. H., Yan, F., Stuart, B. L., Prendini, E., Suwannapoom, C., Dahn, H. A., ... & Che, J. (2020). A combined approach of mitochondrial DNA and anchored nuclear phylogenomics sheds light on unrecognized diversity, phylogeny, and historical biogeography of the torrent frogs, genus *Amolops* (Anura: Ranidae). *Molecular phylogenetics and Evolution*, *148*, 106789.
115	MN953706.1_A_mantzorum_Sinchuan	*Amolops mantzorum*	MN953706.1	Wu et al., 2020	Wu, Y. H., Yan, F., Stuart, B. L., Prendini, E., Suwannapoom, C., Dahn, H. A., ... & Che, J. (2020). A combined approach of mitochondrial DNA and anchored nuclear phylogenomics sheds light on unrecognized diversity, phylogeny, and historical biogeography of the torrent frogs, genus *Amolops* (Anura: Ranidae). *Molecular phylogenetics and Evolution*, *148*, 106789.
116	MN953707.1_A_mantzorum_Sinchuan	*Amolops mantzorum*	MN953707.1	Wu et al., 2020	Wu, Y. H., Yan, F., Stuart, B. L., Prendini, E., Suwannapoom, C., Dahn, H. A., ... & Che, J. (2020). A combined approach of mitochondrial DNA and anchored nuclear phylogenomics sheds light on unrecognized diversity, phylogeny, and historical biogeography of the torrent frogs, genus *Amolops* (Anura: Ranidae). *Molecular phylogenetics and Evolution*, *148*, 106789.
117	MN953764.1_A_xinduqiao_Sinchuan	*Amolops xinduqiao*	MN953764.1	Wu et al., 2020	Wu, Y. H., Yan, F., Stuart, B. L., Prendini, E., Suwannapoom, C., Dahn, H. A., ... & Che, J. (2020). A combined approach of mitochondrial DNA and anchored nuclear phylogenomics sheds light on unrecognized diversity, phylogeny, and historical

					biogeography of the torrent frogs, genus *Amolops* (Anura: Ranidae). *Molecular phylogenetics and Evolution*, *148*, 106789.
118	MN953765.1_A_xinduqiao_Sinchuan	*Amolops xinduqiao*	MN953765.1	Wu et al., 2020	Wu, Y. H., Yan, F., Stuart, B. L., Prendini, E., Suwannapoom, C., Dahn, H. A., ... & Che, J. (2020). A combined approach of mitochondrial DNA and anchored nuclear phylogenomics sheds light on unrecognized diversity, phylogeny, and historical biogeography of the torrent frogs, genus *Amolops* (Anura: Ranidae). *Molecular phylogenetics and Evolution*, *148*, 106789.
119	MN953729.1_A_tuberodepressus_Yunnan	*Amolops tuberodepressus*	MN953729.1	Wu et al., 2020	Wu, Y. H., Yan, F., Stuart, B. L., Prendini, E., Suwannapoom, C., Dahn, H. A., ... & Che, J. (2020). A combined approach of mitochondrial DNA and anchored nuclear phylogenomics sheds light on unrecognized diversity, phylogeny, and historical biogeography of the torrent frogs, genus *Amolops* (Anura: Ranidae). *Molecular phylogenetics and Evolution*, *148*, 106789.
120	MN953730.1_A_tuberodepressus_Yunnan	*Amolops tuberodepressus*	MN953730.1	Wu et al., 2020	Wu, Y. H., Yan, F., Stuart, B. L., Prendini, E., Suwannapoom, C., Dahn, H. A., ... & Che, J. (2020). A combined approach of mitochondrial DNA and anchored nuclear phylogenomics sheds light on unrecognized diversity, phylogeny, and historical biogeography of the torrent frogs, genus *Amolops* (Anura: Ranidae). *Molecular phylogenetics and Evolution*, *148*, 106789.
121	MK604845.1_A_shuichengicus_Guizhou	*Amolops shuichengicus*	MK604845.1	Lyu et al., 2019	Lyu, Z. T., Zeng, Z. C., Wan, H., Yang, J. H., Li, Y. L., Pang, H., & Wang, Y. Y. (2019). A new species of *Amolops* (Anura: Ranidae) from China, with taxonomic comments on *A. liangshanensis* and Chinese populations of *A. marmoratus*. *Zootaxa*, *4609*(2), 247-268.
122	MK604846.1_A_shuichengicus_Guizhou	*Amolops shuichengicus*	MK604846.1	Lyu et al., 2019	Lyu, Z. T., Zeng, Z. C., Wan, H., Yang, J. H., Li, Y. L., Pang, H., & Wang, Y. Y. (2019). A new species of *Amolops* (Anura: Ranidae) from China, with taxonomic comments on *A. liangshanensis* and Chinese populations of *A. marmoratus*. *Zootaxa*, *4609*(2), 247-268.
123	MN953680.1_A_granulosus_Sinchuan	*Amolops granulosus*	MN953680.1	Wu et al., 2020	Wu, Y. H., Yan, F., Stuart, B. L., Prendini, E., Suwannapoom, C., Dahn, H. A., ... & Che, J. (2020). A combined approach of mitochondrial DNA and anchored nuclear phylogenomics sheds light on unrecognized diversity, phylogeny, and historical biogeography of the torrent frogs, genus *Amolops* (Anura: Ranidae). *Molecular phylogenetics and Evolution*, *148*, 106789.
124	MN953681.1_A_granulosus_Sinchuan	*Amolops granulosus*	MN953681.1	Wu et al., 2020	Wu, Y. H., Yan, F., Stuart, B. L., Prendini, E., Suwannapoom, C., Dahn, H. A., ... & Che, J. (2020). A combined approach of mitochondrial DNA and anchored nuclear phylogenomics sheds light on unrecognized diversity, phylogeny, and historical biogeography of the torrent frogs, genus *Amolops* (Anura: Ranidae). *Molecular phylogenetics and Evolution*, *148*, 106789.
125	MN953702.1_A_lifanensis_Sinchuan	*Amolops lifanensis*	MN953702.1	Wu et al., 2020	Wu, Y. H., Yan, F., Stuart, B. L., Prendini, E., Suwannapoom, C., Dahn, H. A., ... & Che, J. (2020). A combined approach of mitochondrial DNA and anchored nuclear phylogenomics sheds light on unrecognized diversity, phylogeny, and historical biogeography of the torrent frogs, genus *Amolops* (Anura: Ranidae). *Molecular phylogenetics and Evolution*, *148*, 106789.
126	MN953703.1_A_lifanensis_Sinchuan	*Amolops lifanensis*	MN953703.1	Wu et al., 2020	Wu, Y. H., Yan, F., Stuart, B. L., Prendini, E., Suwannapoom, C., Dahn, H. A., ... & Che, J. (2020). A combined approach of mitochondrial DNA and anchored nuclear phylogenomics sheds light on unrecognized diversity, phylogeny, and historical biogeography of the torrent frogs, genus *Amolops* (Anura: Ranidae). *Molecular phylogenetics and Evolution*, *148*, 106789.

127	MN953731.1_A_viridimaculatus_1_Yunnan	*Amolops viridimaculatus*	MN953731.1	Wu et al., 2020	Wu, Y. H., Yan, F., Stuart, B. L., Prendini, E., Suwannapoom, C., Dahn, H. A., ... & Che, J. (2020). A combined approach of mitochondrial DNA and anchored nuclear phylogenomics sheds light on unrecognized diversity, phylogeny, and historical biogeography of the torrent frogs, genus *Amolops* (Anura: Ranidae). *Molecular phylogenetics and Evolution*, *148*, 106789.
128	MN953732.1_A_viridimaculatus_1_Yunnan	*Amolops viridimaculatus*	MN953732.1	Wu et al., 2020	Wu, Y. H., Yan, F., Stuart, B. L., Prendini, E., Suwannapoom, C., Dahn, H. A., ... & Che, J. (2020). A combined approach of mitochondrial DNA and anchored nuclear phylogenomics sheds light on unrecognized diversity, phylogeny, and historical biogeography of the torrent frogs, genus *Amolops* (Anura: Ranidae). *Molecular phylogenetics and Evolution*, *148*, 106789.
129	MN953733.1_A_viridimaculatus_1_Yunnan	*Amolops viridimaculatus*	MN953733.1	Wu et al., 2020	Wu, Y. H., Yan, F., Stuart, B. L., Prendini, E., Suwannapoom, C., Dahn, H. A., ... & Che, J. (2020). A combined approach of mitochondrial DNA and anchored nuclear phylogenomics sheds light on unrecognized diversity, phylogeny, and historical biogeography of the torrent frogs, genus *Amolops* (Anura: Ranidae). *Molecular phylogenetics and Evolution*, *148*, 106789.
130	MN953734.1_A_viridimaculatus_1_Yunnan	*Amolops viridimaculatus*	MN953734.1	Wu et al., 2020	Wu, Y. H., Yan, F., Stuart, B. L., Prendini, E., Suwannapoom, C., Dahn, H. A., ... & Che, J. (2020). A combined approach of mitochondrial DNA and anchored nuclear phylogenomics sheds light on unrecognized diversity, phylogeny, and historical biogeography of the torrent frogs, genus *Amolops* (Anura: Ranidae). *Molecular phylogenetics and Evolution*, *148*, 106789.
131	MN953735.1_A_viridimaculatus_1_Yunnan	*Amolops viridimaculatus*	MN953735.1	Wu et al., 2020	Wu, Y. H., Yan, F., Stuart, B. L., Prendini, E., Suwannapoom, C., Dahn, H. A., ... & Che, J. (2020). A combined approach of mitochondrial DNA and anchored nuclear phylogenomics sheds light on unrecognized diversity, phylogeny, and historical biogeography of the torrent frogs, genus *Amolops* (Anura: Ranidae). *Molecular phylogenetics and Evolution*, *148*, 106789.
132	MN953738.1_A_viridimaculatus_1_Yunnan	*Amolops viridimaculatus*	MN953738.1	Wu et al., 2020	Wu, Y. H., Yan, F., Stuart, B. L., Prendini, E., Suwannapoom, C., Dahn, H. A., ... & Che, J. (2020). A combined approach of mitochondrial DNA and anchored nuclear phylogenomics sheds light on unrecognized diversity, phylogeny, and historical biogeography of the torrent frogs, genus *Amolops* (Anura: Ranidae). *Molecular phylogenetics and Evolution*, *148*, 106789.
133	MN953736.1_A_viridimaculatus_2_Yunnan	*Amolops viridimaculatus*	MN953736.1	Wu et al., 2020	Wu, Y. H., Yan, F., Stuart, B. L., Prendini, E., Suwannapoom, C., Dahn, H. A., ... & Che, J. (2020). A combined approach of mitochondrial DNA and anchored nuclear phylogenomics sheds light on unrecognized diversity, phylogeny, and historical biogeography of the torrent frogs, genus *Amolops* (Anura: Ranidae). *Molecular phylogenetics and Evolution*, *148*, 106789.
134	MN953737.1_A_viridimaculatus_2_Yunnan	*Amolops viridimaculatus*	MN953737.1	Wu et al., 2020	Wu, Y. H., Yan, F., Stuart, B. L., Prendini, E., Suwannapoom, C., Dahn, H. A., ... & Che, J. (2020). A combined approach of mitochondrial DNA and anchored nuclear phylogenomics sheds light on unrecognized diversity, phylogeny, and historical biogeography of the torrent frogs, genus *Amolops* (Anura: Ranidae). *Molecular phylogenetics and Evolution*, *148*, 106789.
135	MN953662.1_A_beibengensis_Tibet	*Amolops sp. 2 YW-2020*	MN953662.1	Wu et al., 2020	Wu, Y. H., Yan, F., Stuart, B. L., Prendini, E., Suwannapoom, C., Dahn, H. A., ... & Che, J. (2020). A combined approach of mitochondrial DNA and anchored nuclear phylogenomics sheds light on unrecognized diversity, phylogeny, and historical biogeography of the torrent frogs, genus *Amolops* (Anura: Ranidae). *Molecular phylogenetics and Evolution*, *148*, 106789.

136	MN953663.1_A_beibengensis_Tibet	*Amolops sp. 2 YW-2020*	MN953663.1	Wu et al., 2020	Wu, Y. H., Yan, F., Stuart, B. L., Prendini, E., Suwannapoom, C., Dahn, H. A., ... & Che, J. (2020). A combined approach of mitochondrial DNA and anchored nuclear phylogenomics sheds light on unrecognized diversity, phylogeny, and historical biogeography of the torrent frogs, genus *Amolops* (Anura: Ranidae). *Molecular phylogenetics and Evolution*, *148*, 106789.
137	MN953710.1_A_medogensis_Tibet	*Amolops medogensis*	MN953710.1	Wu et al., 2020	Wu, Y. H., Yan, F., Stuart, B. L., Prendini, E., Suwannapoom, C., Dahn, H. A., ... & Che, J. (2020). A combined approach of mitochondrial DNA and anchored nuclear phylogenomics sheds light on unrecognized diversity, phylogeny, and historical biogeography of the torrent frogs, genus *Amolops* (Anura: Ranidae). *Molecular phylogenetics and Evolution*, *148*, 106789.
138	MN953711.1_A_medogensis_Tibet	*Amolops medogensis*	MN953711.1	Wu et al., 2020	Wu, Y. H., Yan, F., Stuart, B. L., Prendini, E., Suwannapoom, C., Dahn, H. A., ... & Che, J. (2020). A combined approach of mitochondrial DNA and anchored nuclear phylogenomics sheds light on unrecognized diversity, phylogeny, and historical biogeography of the torrent frogs, genus *Amolops* (Anura: Ranidae). *Molecular phylogenetics and Evolution*, *148*, 106789.
139	MN953740.1_A_wangyufani_Tibet	*Amolops sp. 3 YW-2020*	MN953740.1	Wu et al., 2020	Wu, Y. H., Yan, F., Stuart, B. L., Prendini, E., Suwannapoom, C., Dahn, H. A., ... & Che, J. (2020). A combined approach of mitochondrial DNA and anchored nuclear phylogenomics sheds light on unrecognized diversity, phylogeny, and historical biogeography of the torrent frogs, genus *Amolops* (Anura: Ranidae). *Molecular phylogenetics and Evolution*, *148*, 106789.
140	MN953741.1_A_wangyufani_Tibet	*Amolops sp. 3 YW-2020*	MN953741.1	Wu et al., 2020	Wu, Y. H., Yan, F., Stuart, B. L., Prendini, E., Suwannapoom, C., Dahn, H. A., ... & Che, J. (2020). A combined approach of mitochondrial DNA and anchored nuclear phylogenomics sheds light on unrecognized diversity, phylogeny, and historical biogeography of the torrent frogs, genus *Amolops* (Anura: Ranidae). *Molecular phylogenetics and Evolution*, *148*, 106789.
141	MN953682.1_A_gyirongensis_Tibet	*Amolops sp. 4 YW-2020*	MN953682.1	Wu et al., 2020	Wu, Y. H., Yan, F., Stuart, B. L., Prendini, E., Suwannapoom, C., Dahn, H. A., ... & Che, J. (2020). A combined approach of mitochondrial DNA and anchored nuclear phylogenomics sheds light on unrecognized diversity, phylogeny, and historical biogeography of the torrent frogs, genus *Amolops* (Anura: Ranidae). *Molecular phylogenetics and Evolution*, *148*, 106789.
142	MN953686.1_A_gyirongensis_Tibet	*Amolops sp. 4 YW-2020*	MN953686.1	Wu et al., 2020	Wu, Y. H., Yan, F., Stuart, B. L., Prendini, E., Suwannapoom, C., Dahn, H. A., ... & Che, J. (2020). A combined approach of mitochondrial DNA and anchored nuclear phylogenomics sheds light on unrecognized diversity, phylogeny, and historical biogeography of the torrent frogs, genus *Amolops* (Anura: Ranidae). *Molecular phylogenetics and Evolution*, *148*, 106789.
143	MN953684.1_A_gyirongensis_Tibet	*Amolops sp. 4 YW-2020*	MN953684.1	Wu et al., 2020	Wu, Y. H., Yan, F., Stuart, B. L., Prendini, E., Suwannapoom, C., Dahn, H. A., ... & Che, J. (2020). A combined approach of mitochondrial DNA and anchored nuclear phylogenomics sheds light on unrecognized diversity, phylogeny, and historical biogeography of the torrent frogs, genus *Amolops* (Anura: Ranidae). *Molecular phylogenetics and Evolution*, *148*, 106789.
144	MN953683.1_A_gyirongensis_Tibet	*Amolops sp. 4 YW-2020*	MN953683.1	Wu et al., 2020	Wu, Y. H., Yan, F., Stuart, B. L., Prendini, E., Suwannapoom, C., Dahn, H. A., ... & Che, J. (2020). A combined approach of mitochondrial DNA and anchored nuclear phylogenomics sheds light on unrecognized diversity, phylogeny, and historical biogeography of the torrent frogs, genus *Amolops* (Anura: Ranidae). *Molecular phylogenetics and Evolution*, *148*, 106789.

145	MN953685.1_A_gyirongensis_Tibet	*Amolops sp. 4 YW-2020*	MN953685.1	Wu et al., 2020	Wu, Y. H., Yan, F., Stuart, B. L., Prendini, E., Suwannapoom, C., Dahn, H. A., ... & Che, J. (2020). A combined approach of mitochondrial DNA and anchored nuclear phylogenomics sheds light on unrecognized diversity, phylogeny, and historical biogeography of the torrent frogs, genus *Amolops* (Anura: Ranidae). *Molecular phylogenetics and Evolution*, *148*, 106789.
146	MN953712.1_A_sp._5_Nepal	*Amolops sp. 5 YW-2020*	MN953712.1	Wu et al., 2020	Wu, Y. H., Yan, F., Stuart, B. L., Prendini, E., Suwannapoom, C., Dahn, H. A., ... & Che, J. (2020). A combined approach of mitochondrial DNA and anchored nuclear phylogenomics sheds light on unrecognized diversity, phylogeny, and historical biogeography of the torrent frogs, genus *Amolops* (Anura: Ranidae). *Molecular phylogenetics and Evolution*, *148*, 106789.
147	MN953713.1_A_sp._5_Nepal	*Amolops sp. 5 YW-2020*	MN953713.1	Wu et al., 2020	Wu, Y. H., Yan, F., Stuart, B. L., Prendini, E., Suwannapoom, C., Dahn, H. A., ... & Che, J. (2020). A combined approach of mitochondrial DNA and anchored nuclear phylogenomics sheds light on unrecognized diversity, phylogeny, and historical biogeography of the torrent frogs, genus *Amolops* (Anura: Ranidae). *Molecular phylogenetics and Evolution*, *148*, 106789.
148	MN953714.1_A_sp._5_Nepal	*Amolops sp. 5 YW-2020*	MN953714.1	Wu et al., 2020	Wu, Y. H., Yan, F., Stuart, B. L., Prendini, E., Suwannapoom, C., Dahn, H. A., ... & Che, J. (2020). A combined approach of mitochondrial DNA and anchored nuclear phylogenomics sheds light on unrecognized diversity, phylogeny, and historical biogeography of the torrent frogs, genus *Amolops* (Anura: Ranidae). *Molecular phylogenetics and Evolution*, *148*, 106789.
149	MN953654.1_A_afghanus_Yunnan	*Amolops afghanus*	MN953654.1	Wu et al., 2020	Wu, Y. H., Yan, F., Stuart, B. L., Prendini, E., Suwannapoom, C., Dahn, H. A., ... & Che, J. (2020). A combined approach of mitochondrial DNA and anchored nuclear phylogenomics sheds light on unrecognized diversity, phylogeny, and historical biogeography of the torrent frogs, genus *Amolops* (Anura: Ranidae). *Molecular phylogenetics and Evolution*, *148*, 106789.
150	MN953773.1_A_afghanus_Myanmar	*Amolops afghanus*	MN953773.1	Wu et al., 2020	Wu, Y. H., Yan, F., Stuart, B. L., Prendini, E., Suwannapoom, C., Dahn, H. A., ... & Che, J. (2020). A combined approach of mitochondrial DNA and anchored nuclear phylogenomics sheds light on unrecognized diversity, phylogeny, and historical biogeography of the torrent frogs, genus *Amolops* (Anura: Ranidae). *Molecular phylogenetics and Evolution*, *148*, 106789.
151	MN953774.1_A_afghanus_Myanmar	*Amolops afghanus*	MN953774.1	Wu et al., 2020	Wu, Y. H., Yan, F., Stuart, B. L., Prendini, E., Suwannapoom, C., Dahn, H. A., ... & Che, J. (2020). A combined approach of mitochondrial DNA and anchored nuclear phylogenomics sheds light on unrecognized diversity, phylogeny, and historical biogeography of the torrent frogs, genus *Amolops* (Anura: Ranidae). *Molecular phylogenetics and Evolution*, *148*, 106789.
152	MN953692.1_A_indoburmanensis_Myanmar	*Amolops indoburmanensis*	MN953692.1	Wu et al., 2020	Wu, Y. H., Yan, F., Stuart, B. L., Prendini, E., Suwannapoom, C., Dahn, H. A., ... & Che, J. (2020). A combined approach of mitochondrial DNA and anchored nuclear phylogenomics sheds light on unrecognized diversity, phylogeny, and historical biogeography of the torrent frogs, genus *Amolops* (Anura: Ranidae). *Molecular phylogenetics and Evolution*, *148*, 106789.
153	MN953694.1_A_indoburmanensis_Myanmar	*Amolops indoburmanensis*	MN953694.1	Wu et al., 2020	Wu, Y. H., Yan, F., Stuart, B. L., Prendini, E., Suwannapoom, C., Dahn, H. A., ... & Che, J. (2020). A combined approach of mitochondrial DNA and anchored nuclear phylogenomics sheds light on unrecognized diversity, phylogeny, and historical biogeography of the torrent frogs, genus *Amolops* (Anura: Ranidae). *Molecular phylogenetics and Evolution*, *148*, 106789.

154	MN953693.1_A_indoburmanensis_Myanmar	*Amolops indoburmanensis*	MN953693.1	Wu et al., 2020	Wu, Y. H., Yan, F., Stuart, B. L., Prendini, E., Suwannapoom, C., Dahn, H. A., ... & Che, J. (2020). A combined approach of mitochondrial DNA and anchored nuclear phylogenomics sheds light on unrecognized diversity, phylogeny, and historical biogeography of the torrent frogs, genus *Amolops* (Anura: Ranidae). *Molecular phylogenetics and Evolution*, *148*, 106789.
155	MN953708.1_A_marmoratus_Thailand	*Amolops marmoratus*	MN953708.1	Wu et al., 2020	Wu, Y. H., Yan, F., Stuart, B. L., Prendini, E., Suwannapoom, C., Dahn, H. A., ... & Che, J. (2020). A combined approach of mitochondrial DNA and anchored nuclear phylogenomics sheds light on unrecognized diversity, phylogeny, and historical biogeography of the torrent frogs, genus *Amolops* (Anura: Ranidae). *Molecular phylogenetics and Evolution*, *148*, 106789.
156	MN953709.1_A_marmoratus_Thailand	*Amolops marmoratus*	MN953709.1	Wu et al., 2020	Wu, Y. H., Yan, F., Stuart, B. L., Prendini, E., Suwannapoom, C., Dahn, H. A., ... & Che, J. (2020). A combined approach of mitochondrial DNA and anchored nuclear phylogenomics sheds light on unrecognized diversity, phylogeny, and historical biogeography of the torrent frogs, genus *Amolops* (Anura: Ranidae). *Molecular phylogenetics and Evolution*, *148*, 106789.
157	MN953744.1_A_yarlungzanbo_Tibet	*Amolops sp. 6 YW-2020*	MN953744.1	Wu et al., 2020	Wu, Y. H., Yan, F., Stuart, B. L., Prendini, E., Suwannapoom, C., Dahn, H. A., ... & Che, J. (2020). A combined approach of mitochondrial DNA and anchored nuclear phylogenomics sheds light on unrecognized diversity, phylogeny, and historical biogeography of the torrent frogs, genus *Amolops* (Anura: Ranidae). *Molecular phylogenetics and Evolution*, *148*, 106789.
158	MN953745.1_A_yarlungzanbo_Tibet	*Amolops sp. 6 YW-2020*	MN953745.1	Wu et al., 2020	Wu, Y. H., Yan, F., Stuart, B. L., Prendini, E., Suwannapoom, C., Dahn, H. A., ... & Che, J. (2020). A combined approach of mitochondrial DNA and anchored nuclear phylogenomics sheds light on unrecognized diversity, phylogeny, and historical biogeography of the torrent frogs, genus *Amolops* (Anura: Ranidae). *Molecular phylogenetics and Evolution*, *148*, 106789.
159	MN953746.1_A_yarlungzanbo_Tibet	*Amolops sp. 6 YW-2020*	MN953746.1	Wu et al., 2020	Wu, Y. H., Yan, F., Stuart, B. L., Prendini, E., Suwannapoom, C., Dahn, H. A., ... & Che, J. (2020). A combined approach of mitochondrial DNA and anchored nuclear phylogenomics sheds light on unrecognized diversity, phylogeny, and historical biogeography of the torrent frogs, genus *Amolops* (Anura: Ranidae). *Molecular phylogenetics and Evolution*, *148*, 106789.
160	MN953747.1_A_syarlungzanbo_Tibet	*Amolops sp. 6 YW-2020*	MN953747.1	Wu et al., 2020	Wu, Y. H., Yan, F., Stuart, B. L., Prendini, E., Suwannapoom, C., Dahn, H. A., ... & Che, J. (2020). A combined approach of mitochondrial DNA and anchored nuclear phylogenomics sheds light on unrecognized diversity, phylogeny, and historical biogeography of the torrent frogs, genus *Amolops* (Anura: Ranidae). *Molecular phylogenetics and Evolution*, *148*, 106789.
161	MN953750.1_A_sp._7_Nepal	*Amolops sp. 7 YW-2020*	MN953750.1	Wu et al., 2020	Wu, Y. H., Yan, F., Stuart, B. L., Prendini, E., Suwannapoom, C., Dahn, H. A., ... & Che, J. (2020). A combined approach of mitochondrial DNA and anchored nuclear phylogenomics sheds light on unrecognized diversity, phylogeny, and historical biogeography of the torrent frogs, genus *Amolops* (Anura: Ranidae). *Molecular phylogenetics and Evolution*, *148*, 106789.
162	MN953720.1_A_panhai_Thailand	*Amolops panhai*	MN953720.1	Wu et al., 2020	Wu, Y. H., Yan, F., Stuart, B. L., Prendini, E., Suwannapoom, C., Dahn, H. A., ... & Che, J. (2020). A combined approach of mitochondrial DNA and anchored nuclear phylogenomics sheds light on unrecognized diversity, phylogeny, and historical biogeography of the torrent frogs, genus *Amolops* (Anura: Ranidae). *Molecular phylogenetics and Evolution*, *148*, 106789.

163	MN953722.1_A_panhai_Myanmar	*Amolops panhai*	MN953722.1	Wu et al., 2020	Wu, Y. H., Yan, F., Stuart, B. L., Prendini, E., Suwannapoom, C., Dahn, H. A., ... & Che, J. (2020). A combined approach of mitochondrial DNA and anchored nuclear phylogenomics sheds light on unrecognized diversity, phylogeny, and historical biogeography of the torrent frogs, genus *Amolops* (Anura: Ranidae). *Molecular phylogenetics and Evolution*, *148*, 106789.
164	MN953721.1_A_panhai_Thailand	*Amolops panhai*	MN953721.1	Wu et al., 2020	Wu, Y. H., Yan, F., Stuart, B. L., Prendini, E., Suwannapoom, C., Dahn, H. A., ... & Che, J. (2020). A combined approach of mitochondrial DNA and anchored nuclear phylogenomics sheds light on unrecognized diversity, phylogeny, and historical biogeography of the torrent frogs, genus *Amolops* (Anura: Ranidae). *Molecular phylogenetics and Evolution*, *148*, 106789.
165	MN953723.1_A_yatseni_Veitnam	*Amolops yatseni*	MN953723.1	Wu et al., 2020	Wu, Y. H., Yan, F., Stuart, B. L., Prendini, E., Suwannapoom, C., Dahn, H. A., ... & Che, J. (2020). A combined approach of mitochondrial DNA and anchored nuclear phylogenomics sheds light on unrecognized diversity, phylogeny, and historical biogeography of the torrent frogs, genus *Amolops* (Anura: Ranidae). *Molecular phylogenetics and Evolution*, *148*, 106789.
166	MN953758.1_A_yatseni_Guangxi	*Amolops yatseni*	MN953758.1	Wu et al., 2020	Wu, Y. H., Yan, F., Stuart, B. L., Prendini, E., Suwannapoom, C., Dahn, H. A., ... & Che, J. (2020). A combined approach of mitochondrial DNA and anchored nuclear phylogenomics sheds light on unrecognized diversity, phylogeny, and historical biogeography of the torrent frogs, genus *Amolops* (Anura: Ranidae). *Molecular phylogenetics and Evolution*, *148*, 106789.
167	MK263290.1_A_yatseni_Guandong	*Amolops yatseni*	MK263290.1	Lyu et al., 2019	Lyu, Z. T., Huang, L. S., Wang, J., Li, Y. Q., Chen, H. H., Qi, S., & Wang, Y. Y. (2019). Description of two cryptic species of the *Amolopsricketti* group (Anura, Ranidae) from southeastern China. *ZooKeys*, (812), 133.
168	MK263250.1_A_yatseni_Guandong	*Amolops yatseni*	MK263250.1	Lyu et al., 2019	Lyu, Z. T., Huang, L. S., Wang, J., Li, Y. Q., Chen, H. H., Qi, S., & Wang, Y. Y. (2019). Description of two cryptic species of the *Amolopsricketti* group (Anura, Ranidae) from southeastern China. *ZooKeys*, (812), 133.
169	MN953748.1_A_sinensis_Guangxi	*Amolops sinensis*	MN953748.1	Wu et al., 2020	Wu, Y. H., Yan, F., Stuart, B. L., Prendini, E., Suwannapoom, C., Dahn, H. A., ... & Che, J. (2020). A combined approach of mitochondrial DNA and anchored nuclear phylogenomics sheds light on unrecognized diversity, phylogeny, and historical biogeography of the torrent frogs, genus *Amolops* (Anura: Ranidae). *Molecular phylogenetics and Evolution*, *148*, 106789.
170	MN953749.1_A_sinensis_Guangxi	*Amolops sinensis*	MN953749.1	Wu et al., 2020	Wu, Y. H., Yan, F., Stuart, B. L., Prendini, E., Suwannapoom, C., Dahn, H. A., ... & Che, J. (2020). A combined approach of mitochondrial DNA and anchored nuclear phylogenomics sheds light on unrecognized diversity, phylogeny, and historical biogeography of the torrent frogs, genus *Amolops* (Anura: Ranidae). *Molecular phylogenetics and Evolution*, *148*, 106789.
171	MN953775.1_A_sinensis_Hunan	*Amolops sinensis*	MN953775.1	Wu et al., 2020	Wu, Y. H., Yan, F., Stuart, B. L., Prendini, E., Suwannapoom, C., Dahn, H. A., ... & Che, J. (2020). A combined approach of mitochondrial DNA and anchored nuclear phylogenomics sheds light on unrecognized diversity, phylogeny, and historical biogeography of the torrent frogs, genus *Amolops* (Anura: Ranidae). *Molecular phylogenetics and Evolution*, *148*, 106789.
172	MN953763.1_A_sinensis_Guangxi	*Amolops sinensis*	MN953763.1	Wu et al., 2020	Wu, Y. H., Yan, F., Stuart, B. L., Prendini, E., Suwannapoom, C., Dahn, H. A., ... & Che, J. (2020). A combined approach of mitochondrial DNA and anchored nuclear phylogenomics sheds light on unrecognized diversity, phylogeny, and historical

					biogeography of the torrent frogs, genus *Amolops* (Anura: Ranidae). *Molecular phylogenetics and Evolution*, *148*, 106789.
173	MK263248.1_A_albis pinus_Guandong	*Amolops albispinus*	MK263248.1	Lyu et al., 2019	Lyu, Z. T., Huang, L. S., Wang, J., Li, Y. Q., Chen, H. H., Qi, S., & Wang, Y. Y. (2019). Description of two cryptic species of the *Amolopsricketti* group (Anura, Ranidae) from southeastern China. *ZooKeys*, (812), 133.
174	MK263249.1_A_albis pinus_Guandong	*Amolops albispinus*	MK263249.1	Lyu et al., 2019	Lyu, Z. T., Huang, L. S., Wang, J., Li, Y. Q., Chen, H. H., Qi, S., & Wang, Y. Y. (2019). Description of two cryptic species of the *Amolopsricketti* group (Anura, Ranidae) from southeastern China. *ZooKeys*, (812), 133.
175	MK263271.1_A_yun kaiensis_Guandong	*Amolops yunkaiensis*	MK263271.1	Lyu et al., 2019	Lyu, Z. T., Huang, L. S., Wang, J., Li, Y. Q., Chen, H. H., Qi, S., & Wang, Y. Y. (2019). Description of two cryptic species of the *Amolopsricketti* group (Anura, Ranidae) from southeastern China. *ZooKeys*, (812), 133.
176	MK263277.1_A_yun kaiensis_Guandong	*Amolops yunkaiensis*	MK263277.1	Lyu et al., 2019	Lyu, Z. T., Huang, L. S., Wang, J., Li, Y. Q., Chen, H. H., Qi, S., & Wang, Y. Y. (2019). Description of two cryptic species of the *Amolopsricketti* group (Anura, Ranidae) from southeastern China. *ZooKeys*, (812), 133.
177	MN953742.1_A_wuy iensis_Fujian	*Amolops wuyiensis*	MN953742.1	Wu et al., 2020	Wu, Y. H., Yan, F., Stuart, B. L., Prendini, E., Suwannapoom, C., Dahn, H. A., ... & Che, J. (2020). A combined approach of mitochondrial DNA and anchored nuclear phylogenomics sheds light on unrecognized diversity, phylogeny, and historical biogeography of the torrent frogs, genus *Amolops* (Anura: Ranidae). *Molecular phylogenetics and Evolution*, *148*, 106789.
178	MN953760.1_A_wuy iensis_Zhejiang	*Amolops wuyiensis*	MN953760.1	Wu et al., 2020	Wu, Y. H., Yan, F., Stuart, B. L., Prendini, E., Suwannapoom, C., Dahn, H. A., ... & Che, J. (2020). A combined approach of mitochondrial DNA and anchored nuclear phylogenomics sheds light on unrecognized diversity, phylogeny, and historical biogeography of the torrent frogs, genus *Amolops* (Anura: Ranidae). *Molecular phylogenetics and Evolution*, *148*, 106789.
179	MN953761.1_A_wuy iensis_Zhejiang	*Amolops wuyiensis*	MN953761.1	Wu et al., 2020	Wu, Y. H., Yan, F., Stuart, B. L., Prendini, E., Suwannapoom, C., Dahn, H. A., ... & Che, J. (2020). A combined approach of mitochondrial DNA and anchored nuclear phylogenomics sheds light on unrecognized diversity, phylogeny, and historical biogeography of the torrent frogs, genus *Amolops* (Anura: Ranidae). *Molecular phylogenetics and Evolution*, *148*, 106789.
180	MN953743.1_A_rick etti_China	*Amolops ricketti*	MN953743.1	Wu et al., 2020	Wu, Y. H., Yan, F., Stuart, B. L., Prendini, E., Suwannapoom, C., Dahn, H. A., ... & Che, J. (2020). A combined approach of mitochondrial DNA and anchored nuclear phylogenomics sheds light on unrecognized diversity, phylogeny, and historical biogeography of the torrent frogs, genus *Amolops* (Anura: Ranidae). *Molecular phylogenetics and Evolution*, *148*, 106789.
181	MN953759.1_A_rick etti_China	*Amolops ricketti*	MN953759.1	Wu et al., 2020	Wu, Y. H., Yan, F., Stuart, B. L., Prendini, E., Suwannapoom, C., Dahn, H. A., ... & Che, J. (2020). A combined approach of mitochondrial DNA and anchored nuclear phylogenomics sheds light on unrecognized diversity, phylogeny, and historical biogeography of the torrent frogs, genus *Amolops* (Anura: Ranidae). *Molecular phylogenetics and Evolution*, *148*, 106789.
182	MN953675.1_A_daiy unensis_Fujian	*Amolops daiyunensis*	MN953675.1	Wu et al., 2020	Wu, Y. H., Yan, F., Stuart, B. L., Prendini, E., Suwannapoom, C., Dahn, H. A., ... & Che, J. (2020). A combined approach of mitochondrial DNA and anchored nuclear phylogenomics sheds light on unrecognized diversity, phylogeny, and historical biogeography of the torrent frogs, genus *Amolops* (Anura: Ranidae). *Molecular phylogenetics and Evolution*, *148*, 106789.

183	MN953676.1_A_daiyunensis_Fujian	*Amolops daiyunensis*	MN953676.1	Wu et al., 2020	Wu, Y. H., Yan, F., Stuart, B. L., Prendini, E., Suwannapoom, C., Dahn, H. A., ... & Che, J. (2020). A combined approach of mitochondrial DNA and anchored nuclear phylogenomics sheds light on unrecognized diversity, phylogeny, and historical biogeography of the torrent frogs, genus *Amolops* (Anura: Ranidae). *Molecular phylogenetics and Evolution*, *148*, 106789.
184	MN953677.1_A_daiyunensis_Fujian	*Amolops daiyunensis*	MN953677.1	Wu et al., 2020	Wu, Y. H., Yan, F., Stuart, B. L., Prendini, E., Suwannapoom, C., Dahn, H. A., ... & Che, J. (2020). A combined approach of mitochondrial DNA and anchored nuclear phylogenomics sheds light on unrecognized diversity, phylogeny, and historical biogeography of the torrent frogs, genus *Amolops* (Anura: Ranidae). *Molecular phylogenetics and Evolution*, *148*, 106789.
185	MN953689.1_A_hongkongensis_Hong_Kong	*Amolops hongkongensis*	MN953689.1	Wu et al., 2020	Wu, Y. H., Yan, F., Stuart, B. L., Prendini, E., Suwannapoom, C., Dahn, H. A., ... & Che, J. (2020). A combined approach of mitochondrial DNA and anchored nuclear phylogenomics sheds light on unrecognized diversity, phylogeny, and historical biogeography of the torrent frogs, genus *Amolops* (Anura: Ranidae). *Molecular phylogenetics and Evolution*, *148*, 106789.
186	MN953691.1_A_hongkongensis_Hong_Kong	*Amolops hongkongensis*	MN953691.1	Wu et al., 2020	Wu, Y. H., Yan, F., Stuart, B. L., Prendini, E., Suwannapoom, C., Dahn, H. A., ... & Che, J. (2020). A combined approach of mitochondrial DNA and anchored nuclear phylogenomics sheds light on unrecognized diversity, phylogeny, and historical biogeography of the torrent frogs, genus *Amolops* (Anura: Ranidae). *Molecular phylogenetics and Evolution*, *148*, 106789.
187	MN953690.1_A_hongkongensis_Hong_Kong	*Amolops hongkongensis*	MN953690.1	Wu et al., 2020	Wu, Y. H., Yan, F., Stuart, B. L., Prendini, E., Suwannapoom, C., Dahn, H. A., ... & Che, J. (2020). A combined approach of mitochondrial DNA and anchored nuclear phylogenomics sheds light on unrecognized diversity, phylogeny, and historical biogeography of the torrent frogs, genus *Amolops* (Anura: Ranidae). *Molecular phylogenetics and Evolution*, *148*, 106789.
188	MN953687.1_A_hainanensis_Hainan	*Amolops hainanensis*	MN953687.1	Wu et al., 2020	Wu, Y. H., Yan, F., Stuart, B. L., Prendini, E., Suwannapoom, C., Dahn, H. A., ... & Che, J. (2020). A combined approach of mitochondrial DNA and anchored nuclear phylogenomics sheds light on unrecognized diversity, phylogeny, and historical biogeography of the torrent frogs, genus *Amolops* (Anura: Ranidae). *Molecular phylogenetics and Evolution*, *148*, 106789.
189	MN953688.1_A_hainanensis_Hainan	*Amolops hainanensis*	MN953688.1	Wu et al., 2020	Wu, Y. H., Yan, F., Stuart, B. L., Prendini, E., Suwannapoom, C., Dahn, H. A., ... & Che, J. (2020). A combined approach of mitochondrial DNA and anchored nuclear phylogenomics sheds light on unrecognized diversity, phylogeny, and historical biogeography of the torrent frogs, genus *Amolops* (Anura: Ranidae). *Molecular phylogenetics and Evolution*, *148*, 106789.
190	EF453744.1_A_torrentis_Hainan	*Amolops torrentis*	EF453744.1	Cai & Zhang (submitted 2007)	Cai, H., & Zhang, Y. (Unpublished). A Re-analysis of the Phylogeny of the Genus *Amolops* (Anura, Ranidae), with Particular Reference to the Systematic Position of Wurana tormota.
191	MN953728.1_A_torrentis_Hainan	*Amolops torrentis*	MN953728.1	Wu et al., 2020	Wu, Y. H., Yan, F., Stuart, B. L., Prendini, E., Suwannapoom, C., Dahn, H. A., ... & Che, J. (2020). A combined approach of mitochondrial DNA and anchored nuclear phylogenomics sheds light on unrecognized diversity, phylogeny, and historical biogeography of the torrent frogs, genus *Amolops* (Anura: Ranidae). *Molecular phylogenetics and Evolution*, *148*, 106789.
192	MN953726.1_A_spinapectoralis_Veitnam	*Amolops spinapectoralis*	MN953726.1	Wu et al., 2020	Wu, Y. H., Yan, F., Stuart, B. L., Prendini, E., Suwannapoom, C., Dahn, H. A., ... & Che, J. (2020). A combined approach of mitochondrial DNA and anchored nuclear

					phylogenomics sheds light on unrecognized diversity, phylogeny, and historical biogeography of the torrent frogs, genus *Amolops* (Anura: Ranidae). *Molecular phylogenetics and Evolution*, *148*, 106789.
193	MN953771.1_A_spinapectoralis_Veitnam	*Amolops spinapectoralis*	MN953771.1	Wu et al., 2020	Wu, Y. H., Yan, F., Stuart, B. L., Prendini, E., Suwannapoom, C., Dahn, H. A., ... & Che, J. (2020). A combined approach of mitochondrial DNA and anchored nuclear phylogenomics sheds light on unrecognized diversity, phylogeny, and historical biogeography of the torrent frogs, genus *Amolops* (Anura: Ranidae). *Molecular phylogenetics and Evolution*, *148*, 106789.
194	MN953727.1_A_spinapectoralis_Veitnam	*Amolops spinapectoralis*	MN953727.1	Wu et al., 2020	Wu, Y. H., Yan, F., Stuart, B. L., Prendini, E., Suwannapoom, C., Dahn, H. A., ... & Che, J. (2020). A combined approach of mitochondrial DNA and anchored nuclear phylogenomics sheds light on unrecognized diversity, phylogeny, and historical biogeography of the torrent frogs, genus *Amolops* (Anura: Ranidae). *Molecular phylogenetics and Evolution*, *148*, 106789.
195	MN953770.1_A_spinapectoralis_Veitnam	*Amolops spinapectoralis*	MN953770.1	Wu et al., 2020	Wu, Y. H., Yan, F., Stuart, B. L., Prendini, E., Suwannapoom, C., Dahn, H. A., ... & Che, J. (2020). A combined approach of mitochondrial DNA and anchored nuclear phylogenomics sheds light on unrecognized diversity, phylogeny, and historical biogeography of the torrent frogs, genus *Amolops* (Anura: Ranidae). *Molecular phylogenetics and Evolution*, *148*, 106789.
196	MN953769.1_A_spinapectoralis_Veitnam	*Amolops spinapectoralis*	MN953769.1	Wu et al., 2020	Wu, Y. H., Yan, F., Stuart, B. L., Prendini, E., Suwannapoom, C., Dahn, H. A., ... & Che, J. (2020). A combined approach of mitochondrial DNA and anchored nuclear phylogenomics sheds light on unrecognized diversity, phylogeny, and historical biogeography of the torrent frogs, genus *Amolops* (Anura: Ranidae). *Molecular phylogenetics and Evolution*, *148*, 106789.
197	MN953772.1_A_spinapectoralis_Veitnam	*Amolops spinapectoralis*	MN953772.1	Wu et al., 2020	Wu, Y. H., Yan, F., Stuart, B. L., Prendini, E., Suwannapoom, C., Dahn, H. A., ... & Che, J. (2020). A combined approach of mitochondrial DNA and anchored nuclear phylogenomics sheds light on unrecognized diversity, phylogeny, and historical biogeography of the torrent frogs, genus *Amolops* (Anura: Ranidae). *Molecular phylogenetics and Evolution*, *148*, 106789.
198	MN953768.1_A_spinapectoralis_Veitnam	*Amolops spinapectoralis*	MN953768.1	Wu et al., 2020	Wu, Y. H., Yan, F., Stuart, B. L., Prendini, E., Suwannapoom, C., Dahn, H. A., ... & Che, J. (2020). A combined approach of mitochondrial DNA and anchored nuclear phylogenomics sheds light on unrecognized diversity, phylogeny, and historical biogeography of the torrent frogs, genus *Amolops* (Anura: Ranidae). *Molecular phylogenetics and Evolution*, *148*, 106789.
199	MN953672.1_A_cremnobatus_Veitnam	*Amolops cremnobatus*	MN953672.1	Wu et al., 2020	Wu, Y. H., Yan, F., Stuart, B. L., Prendini, E., Suwannapoom, C., Dahn, H. A., ... & Che, J. (2020). A combined approach of mitochondrial DNA and anchored nuclear phylogenomics sheds light on unrecognized diversity, phylogeny, and historical biogeography of the torrent frogs, genus *Amolops* (Anura: Ranidae). *Molecular phylogenetics and Evolution*, *148*, 106789.
200	MN953673.1_A_cremnobatus_Veitnam	*Amolops cremnobatus*	MN953673.1	Wu et al., 2020	Wu, Y. H., Yan, F., Stuart, B. L., Prendini, E., Suwannapoom, C., Dahn, H. A., ... & Che, J. (2020). A combined approach of mitochondrial DNA and anchored nuclear phylogenomics sheds light on unrecognized diversity, phylogeny, and historical biogeography of the torrent frogs, genus *Amolops* (Anura: Ranidae). *Molecular phylogenetics and Evolution*, *148*, 106789.

201	FJ417143.2_A_cremnobatus_Laos	*Amolops shihaitaoi*	FJ417143.2	Stuart et al., 2010	Stuart, B. L., Bain, R. H., Phimmachak, S., & Spence, K. (2010). Phylogenetic systematics of the *Amolops monticola* group (Amphibia: Ranidae), with description of a new species from northwestern Laos. *Herpetologica*, *66*(1), 52-66.
202	MN953674.1_A_cremnobatus_Thailand	*Amolops cremnobatus*	MN953674.1	Wu et al., 2020	Wu, Y. H., Yan, F., Stuart, B. L., Prendini, E., Suwannapoom, C., Dahn, H. A., ... & Che, J. (2020). A combined approach of mitochondrial DNA and anchored nuclear phylogenomics sheds light on unrecognized diversity, phylogeny, and historical biogeography of the torrent frogs, genus *Amolops* (Anura: Ranidae). *Molecular phylogenetics and Evolution*, *148*, 106789.
203	MF061721.1_A_gerutu_Malaysia	*Amolops larutensis*	MF061721.1	Chan (submitted 2017)	Chan, K.O. (Unpublished). Biodiversity Institute, university of kansas, 1012 Emery Rd Apt B13, Lawrence, KS 66044, USA.
204	MF061745.1_A_australis_Malaysia	*Amolops larutensis*	MF061745.1	Chan (submitted 2017)	Chan, K.O. (Unpublished). Biodiversity Institute, university of kansas, 1012 Emery Rd Apt B13, Lawrence, KS 66044, USA.
205	MF061749.1_A_larutensis_Malaysia	*Amolops larutensis*	MF061749.1	Chan (submitted 2017)	Chan, K.O. (Unpublished). Biodiversity Institute, university of kansas, 1012 Emery Rd Apt B13, Lawrence, KS 66044, USA.
206	MZ229772.1_A_adicola_Arunachal	*Amolops adicola*	MZ229772.1	Patel et al., 2021	Patel, N. G., Garg, S., Das, A., Stuart, B. L., & Biju, S. D. (2021). Phylogenetic position of the poorly known montane cascade frog *Amolops monticola* (Ranidae) and description of a new closely related species from Northeast India. *Journal of Natural History*, *55*(21-22), 1403-1440.
207	MZ229773.1_A_monticola_Sikkim	*Amolops adicola*	MZ229773.1	Patel et al., 2021	Patel, N. G., Garg, S., Das, A., Stuart, B. L., & Biju, S. D. (2021). Phylogenetic position of the poorly known montane cascade frog *Amolops monticola* (Ranidae) and description of a new closely related species from Northeast India. *Journal of Natural History*, *55*(21-22), 1403-1440.
208	MZ229774.1_A_kohimaensis_Nagaland	*Amolops adicola*	MZ229774.1	Patel et al., 2021	Patel, N. G., Garg, S., Das, A., Stuart, B. L., & Biju, S. D. (2021). Phylogenetic position of the poorly known montane cascade frog *Amolops monticola* (Ranidae) and description of a new closely related species from Northeast India. *Journal of Natural History*, *55*(21-22), 1403-1440.
209	MT124507.1_A_mahabharatensis_Chitwan_Nepal	*Amolops mahabharatensis*	MT124507.1	Khatiwada et al., 2020	Khatiwada, J. R., Shu, G., Wang, B., Zhao, T., Xie, F., & Jiang, J. (2020). Description of a new species of *Amolops* Cope, 1865 (Amphibia: Ranidae) from Nepal and nomenclatural validation of *Amolops nepalicus* Yang, 1991. *Asian Herpetological Research*, *11*(2), 71-95.
210	MT124508.1_A_mahabharatensis_Chitwan_Nepal	*Amolops mahabharatensis*	MT124508.1	Khatiwada et al., 2020	Khatiwada, J. R., Shu, G., Wang, B., Zhao, T., Xie, F., & Jiang, J. (2020). Description of a new species of *Amolops* Cope, 1865 (Amphibia: Ranidae) from Nepal and nomenclatural validation of *Amolops nepalicus* Yang, 1991. *Asian Herpetological Research*, *11*(2), 71-95.
211	MT124509.1_A_mahabharatensis_Chitwan_Nepal	*Amolops mahabharatensis*	MT124509.1	Khatiwada et al., 2020	Khatiwada, J. R., Shu, G., Wang, B., Zhao, T., Xie, F., & Jiang, J. (2020). Description of a new species of *Amolops* Cope, 1865 (Amphibia: Ranidae) from Nepal and nomenclatural validation of *Amolops nepalicus* Yang, 1991. *Asian Herpetological Research*, *11*(2), 71-95.
212	MT124510.1_A_mahabharatensis_Chitwan_Nepal	*Amolops mahabharatensis*	MT124510.1	Khatiwada et al., 2020	Khatiwada, J. R., Shu, G., Wang, B., Zhao, T., Xie, F., & Jiang, J. (2020). Description of a new species of *Amolops* Cope, 1865 (Amphibia: Ranidae) from Nepal and nomenclatural validation of *Amolops nepalicus* Yang, 1991. *Asian Herpetological Research*, *11*(2), 71-95.
213	MT124511.1_A_mahabharatensis_Kaski_Nepal	*Amolops mahabharatensis*	MT124511.1	Khatiwada et al., 2020	Khatiwada, J. R., Shu, G., Wang, B., Zhao, T., Xie, F., & Jiang, J. (2020). Description of a new species of *Amolops* Cope, 1865 (Amphibia: Ranidae) from Nepal and

					nomenclatural validation of *Amolops nepalicus* Yang, 1991. *Asian Herpetological Research*, *11*(2), 71-95.
214	MT124512.1_A_mahabharatensis_Kaski_Nepal	*Amolops mahabharatensis*	MT124512.1	Khatiwada et al., 2020	Khatiwada, J. R., Shu, G., Wang, B., Zhao, T., Xie, F., & Jiang, J. (2020). Description of a new species of *Amolops* Cope, 1865 (Amphibia: Ranidae) from Nepal and nomenclatural validation of *Amolops nepalicus* Yang, 1991. *Asian Herpetological Research*, *11*(2), 71-95.
215	MT124513.1_A_mahabharatensis_Sunsari_Nepal	*Amolops mahabharatensis*	MT124513.1	Khatiwada et al., 2020	Khatiwada, J. R., Shu, G., Wang, B., Zhao, T., Xie, F., & Jiang, J. (2020). Description of a new species of *Amolops* Cope, 1865 (Amphibia: Ranidae) from Nepal and nomenclatural validation of *Amolops nepalicus* Yang, 1991. *Asian Herpetological Research*, *11*(2), 71-95.
216	MT124514.1_A_mahabharatensis_Sunsari_Nepal	*Amolops mahabharatensis*	MT124514.1	Khatiwada et al., 2020	Khatiwada, J. R., Shu, G., Wang, B., Zhao, T., Xie, F., & Jiang, J. (2020). Description of a new species of *Amolops* Cope, 1865 (Amphibia: Ranidae) from Nepal and nomenclatural validation of *Amolops nepalicus* Yang, 1991. *Asian Herpetological Research*, *11*(2), 71-95.
217	MT124515.1_A_mahabharatensis_Darchula_Nepal	*Amolops mahabharatensis*	MT124515.1	Khatiwada et al., 2020	Khatiwada, J. R., Shu, G., Wang, B., Zhao, T., Xie, F., & Jiang, J. (2020). Description of a new species of *Amolops* Cope, 1865 (Amphibia: Ranidae) from Nepal and nomenclatural validation of *Amolops nepalicus* Yang, 1991. *Asian Herpetological Research*, *11*(2), 71-95.
218	MT124516.1_A_mahabharatensis_Darchula_Nepal	*Amolops mahabharatensis*	MT124516.1	Khatiwada et al., 2020	Khatiwada, J. R., Shu, G., Wang, B., Zhao, T., Xie, F., & Jiang, J. (2020). Description of a new species of *Amolops* Cope, 1865 (Amphibia: Ranidae) from Nepal and nomenclatural validation of *Amolops nepalicus* Yang, 1991. *Asian Herpetological Research*, *11*(2), 71-95.
219	JF794431.1_A_afganus_Kachin_Myanmar	*Amolops marmoratus*	JF794431.1	Dever et al., 2012	Dever, J. A., Fuiten, A. M., Konu, Ö., & Wilkinson, J. A. (2012). Cryptic torrent frogs of Myanmar: an examination of the *Amolops marmoratus* species complex with the resurrection of *Amolops afghanus* and the identification of a new species. *Ichthyology & Herpetology*, *2012*(1), 57-76.
220	KX507312.1_A_albispinus_Guandong_China	*Amolops sp. YYW-2016*	KX507312.1	Sung et al., 2016	Sung, Y. H., Hu, P., Wang, J., Liu, H. J., & Wang, Y. Y. (2016). A new species of *Amolops* (Anura: Ranidae) from southern China. *Zootaxa*, *4170*(3), 525-538.
221	MF061745.1_A_larutensis_Johor_Malaysia	*Amolops larutensis*	MF061745.1	Chan (submitted 2017)	Chan, K.O. (Unpublished). Biodiversity Institute, university of kansas, 1012 Emery Rd Apt B13, Lawrence, KS 66044, USA.
222	FJ417127.2_A_bellulus_Yunnan_China	*Amolops bellulus*	FJ417127.2	Stuart et al., 2010	Stuart, B. L., Bain, R. H., Phimmachak, S., & Spence, K. (2010). Phylogenetic systematics of the *Amolops monticola* group (Amphibia: Ranidae), with description of a new species from northwestern Laos. *Herpetologica*, *66*(1), 52-66.
223	MK604877.1_A_chayuensis_Xizang_China	*Amolops chayuensis*	MK604877.1	Lyu et al., 2019	Lyu, Z. T., Zeng, Z. C., Wan, H., Yang, J. H., Li, Y. L., Pang, H., & Wang, Y. Y. (2019). A new species of *Amolops* (Anura: Ranidae) from China, with taxonomic comments on *A. liangshanensis* and Chinese populations of *A. marmoratus*. *Zootaxa*, *4609*(2), 247-268.
224	DQ204477.1_A_cremnobatus_Khe_Moi_Vietnam	*Amolops cremnobatus*	DQ204477.1	Ngo et al., 2006	Ngo, A., Murphy, R., Liu, W., Lathrop, A., & Orlov, N. (2006). The phylogenetic relationships of the Chinese and Vietnamese waterfall frogs of the genus *Amolops*. *Amphibia-reptilia*, *27*(1), 81-92.
225	FJ417144.2_A_cucae_Van_Ban_Vietnam	*Amolops cucae*	FJ417144.2	Stuart et al., 2010	Stuart, B. L., Bain, R. H., Phimmachak, S., & Spence, K. (2010). Phylogenetic systematics of the *Amolops monticola* group (Amphibia: Ranidae), with description of a new species from northwestern Laos. *Herpetologica*, *66*(1), 52-66.

226	KX507306.1_A_daiyunensis_Fujian_China	*Amolops daiyunensis*	KX507306.1	Sung et al., 2016	Sung, Y. H., Hu, P., Wang, J., Liu, H. J., & Wang, Y. Y. (2016). A new species of *Amolops* (Anura: Ranidae) from southern China. *Zootaxa*, *4170*(3), 525-538.
227	MT124517.1_A_formosus_Sankhuwasabha_Nepal	*Amolops formosus*	MT124517.1	Khatiwada et al., 2020	Khatiwada, J. R., Shu, G., Wang, B., Zhao, T., Xie, F., & Jiang, J. (2020). Description of a new species of *Amolops* Cope, 1865 (Amphibia: Ranidae) from Nepal and nomenclatural validation of *Amolops nepalicus* Yang, 1991. *Asian Herpetological Research*, *11*(2), 71-95.
228	MT124518.1_A_formosus_Sankhuwasabha_Nepal	*Amolops formosus*	MT124518.1	Khatiwada et al., 2020	Khatiwada, J. R., Shu, G., Wang, B., Zhao, T., Xie, F., & Jiang, J. (2020). Description of a new species of *Amolops* Cope, 1865 (Amphibia: Ranidae) from Nepal and nomenclatural validation of *Amolops nepalicus* Yang, 1991. *Asian Herpetological Research*, *11*(2), 71-95.
229	MT124519.1_A_formosus_Taplejung_Nepal	*Amolops formosus*	MT124519.1	Khatiwada et al., 2020	Khatiwada, J. R., Shu, G., Wang, B., Zhao, T., Xie, F., & Jiang, J. (2020). Description of a new species of *Amolops* Cope, 1865 (Amphibia: Ranidae) from Nepal and nomenclatural validation of *Amolops nepalicus* Yang, 1991. *Asian Herpetological Research*, *11*(2), 71-95.
230	MT124520.1_A_formosus_Taplejung_Nepal	*Amolops formosus*	MT124520.1	Khatiwada et al., 2020	Khatiwada, J. R., Shu, G., Wang, B., Zhao, T., Xie, F., & Jiang, J. (2020). Description of a new species of *Amolops* Cope, 1865 (Amphibia: Ranidae) from Nepal and nomenclatural validation of *Amolops nepalicus* Yang, 1991. *Asian Herpetological Research*, *11*(2), 71-95.
231	MF061741.1_A_larutensis_Perak_Malaysia	*Amolops larutensis*	MF061741.1	Chan (submitted 2017)	Chan, K.O. (Unpublished). Biodiversity Institute, university of kansas, 1012 Emery Rd Apt B13, Lawrence, KS 66044, USA.
232	AB211481.1_A_lifanensis_Sichuan_China	*Amolops granulosus*	AB211481.1	Matsui et al., 2006	Matsui, M., Shimada, T., Liu, W. Z., Maryati, M., Khonsue, W., & Orlov, N. (2006). Phylogenetic relationships of Oriental torrent frogs in the genus *Amolops* and its allies (Amphibia, Anura, Ranidae). *Molecular Phylogenetics and Evolution*, *38*(3), 659-666.
233	KX507320.1_A_hainanensis_Hainan_China	*Amolops hainanensis*	KX507320.1	Sung et al., 2016	Sung, Y. H., Hu, P., Wang, J., Liu, H. J., & Wang, Y. Y. (2016). A new species of *Amolops* (Anura: Ranidae) from southern China. *Zootaxa*, *4170*(3), 525-538.
234	KX507317.1_A_hongkongensis_Hong_Kong_China	*Amolops hongkongensis*	KX507317.1	Sung et al., 2016	Sung, Y. H., Hu, P., Wang, J., Liu, H. J., & Wang, Y. Y. (2016). A new species of *Amolops* (Anura: Ranidae) from southern China. *Zootaxa*, *4170*(3), 525-538.
235	JF794460.1_A_indoburmanensis_Rakhine_Myanmar	*Amolops indoburmanensis*	JF794460.1	Dever et al., 2012	Dever, J. A., Fuiten, A. M., Konu, Ö., & Wilkinson, J. A. (2012). Cryptic torrent frogs of Myanmar: an examination of the *Amolops marmoratus* species complex with the resurrection of *Amolops afghanus* and the identification of a new species. *Ichthyology & Herpetology*, *2012*(1), 57-76.
236	EF453741.1_A_jinjiangensis_Deqing_China	*Amolops jinjiangensis*	EF453741.1	Cai & Zhang (submitted 2007)	Cai, H., & Zhang, Y. (Unpublished). A Re-analysis of the Phylogeny of the Genus *Amolops* (Anura, Ranidae), with Particular Reference to the Systematic Position of Wurana tormota.
237	EF453742.1_A_mantzorum_Sichuan_China	*Amolops kangtingensis*	EF453742.1	Cai & Zhang (submitted 2007)	Cai, H., & Zhang, Y. (Unpublished). A Re-analysis of the Phylogeny of the Genus *Amolops* (Anura, Ranidae), with Particular Reference to the Systematic Position of Wurana tormota.
238	AB211482.1_A_lifanensis_Sichuan_China	*Amolops lifanensis*	AB211482.1	Matsui et al., 2006	Matsui, M., Shimada, T., Liu, W. Z., Maryati, M., Khonsue, W., & Orlov, N. (2006). Phylogenetic relationships of Oriental torrent frogs in the genus *Amolops* and its allies (Amphibia, Anura, Ranidae). *Molecular Phylogenetics and Evolution*, *38*(3), 659-666.
239	AB211478.1_A_loloensis_Sichuan_China	*Amolops loloensis*	AB211478.1	Matsui et al., 2006	Matsui, M., Shimada, T., Liu, W. Z., Maryati, M., Khonsue, W., & Orlov, N. (2006). Phylogenetic relationships of Oriental torrent frogs in the genus *Amolops* and its allies (Amphibia, Anura, Ranidae). *Molecular Phylogenetics and Evolution*, *38*(3), 659-666.

240	EF453743.1_A_loloensis_Sichuan_China	*Amolops liangshanensis*	EF453743.1	Cai & Zhang (submitted 2007)	Cai, H., & Zhang, Y. (Unpublished). A Re-analysis of the Phylogeny of the Genus *Amolops* (Anura, Ranidae), with Particular Reference to the Systematic Position of Wurana tormota.
241	AB211479.1_A_mantzorum_Sichuan_China	*Amolops mantzorum*	AB211479.1	Matsui et al., 2006	Matsui, M., Shimada, T., Liu, W. Z., Maryati, M., Khonsue, W., & Orlov, N. (2006). Phylogenetic relationships of Oriental torrent frogs in the genus *Amolops* and its allies (Amphibia, Anura, Ranidae). *Molecular Phylogenetics and Evolution*, *38*(3), 659-666.
242	JF794453.1_A_marmoratus_Myanmar	*Amolops marmoratus*	JF794453.1	Dever et al., 2012	Dever, J. A., Fuiten, A. M., Konu, Ö., & Wilkinson, J. A. (2012). Cryptic torrent frogs of Myanmar: an examination of the *Amolops marmoratus* species complex with the resurrection of *Amolops afghanus* and the identification of a new species. *Ichthyology & Herpetology*, *2012*(1), 57-76.
243	JF794454.1_A_marmoratus_Myanmar	*Amolops marmoratus*	JF794454.1	Dever et al., 2012	Dever, J. A., Fuiten, A. M., Konu, Ö., & Wilkinson, J. A. (2012). Cryptic torrent frogs of Myanmar: an examination of the *Amolops marmoratus* species complex with the resurrection of *Amolops afghanus* and the identification of a new species. *Ichthyology & Herpetology*, *2012*(1), 57-76.
244	MK573813.1_A_medogensis_Xizang_China	*Amolops medogensis*	MK573813.1	Lyu et al., 2019	Lyu, Z. T., Zeng, Z. C., Wan, H., Yang, J. H., Li, Y. L., Pang, H., & Wang, Y. Y. (2019). A new species of *Amolops* (Anura: Ranidae) from China, with taxonomic comments on *A. liangshanensis* and Chinese populations of *A. marmoratus*. *Zootaxa*, *4609*(2), 247-268.
245	KR827703.1_A_mengyangensis_Sapa_Vietnam	*Amolops mengyangensis*	KR827703.1	Grosjean et al., 2015	Grosjean, S., Ohler, A., Chuaynkern, Y., Cruaud, C., & Hassanin, A. (2015). Improving biodiversity assessment of anuran amphibians using DNA barcoding of tadpoles. Case studies from Southeast Asia. *Comptes Rendus. Biologies*, *338*(5), 351-361.
246	MT124521.1_A_nepalicus_Sankhuwasabha_Nepal	*Amolops nepalicus*	MT124521.1	Khatiwada et al., 2020	Khatiwada, J. R., Shu, G., Wang, B., Zhao, T., Xie, F., & Jiang, J. (2020). Description of a new species of *Amolops* Cope, 1865 (Amphibia: Ranidae) from Nepal and nomenclatural validation of *Amolops nepalicus* Yang, 1991. *Asian Herpetological Research*, *11*(2), 71-95.
247	MT124522.1_A_nepalicus_Sankhuwasabha_Nepal	*Amolops nepalicus*	MT124522.1	Khatiwada et al., 2020	Khatiwada, J. R., Shu, G., Wang, B., Zhao, T., Xie, F., & Jiang, J. (2020). Description of a new species of *Amolops* Cope, 1865 (Amphibia: Ranidae) from Nepal and nomenclatural validation of *Amolops nepalicus* Yang, 1991. *Asian Herpetological Research*, *11*(2), 71-95.
248	MT124523.1_A_nepalicus_Sankhuwasabha_Nepal	*Amolops nepalicus*	MT124523.1	Khatiwada et al., 2020	Khatiwada, J. R., Shu, G., Wang, B., Zhao, T., Xie, F., & Jiang, J. (2020). Description of a new species of *Amolops* Cope, 1865 (Amphibia: Ranidae) from Nepal and nomenclatural validation of *Amolops nepalicus* Yang, 1991. *Asian Herpetological Research*, *11*(2), 71-95.
249	MT124524.1_A_nepalicus_Sankhuwasabha_Nepal	*Amolops nepalicus*	MT124524.1	Khatiwada et al., 2020	Khatiwada, J. R., Shu, G., Wang, B., Zhao, T., Xie, F., & Jiang, J. (2020). Description of a new species of *Amolops* Cope, 1865 (Amphibia: Ranidae) from Nepal and nomenclatural validation of *Amolops nepalicus* Yang, 1991. *Asian Herpetological Research*, *11*(2), 71-95.
250	MK573815.1_A_nyingchiensis_Xizang_China	*Amolops nyingchiensis*	MK573815.1	Lyu et al., 2019	Lyu, Z. T., Zeng, Z. C., Wan, H., Yang, J. H., Li, Y. L., Pang, H., & Wang, Y. Y. (2019). A new species of *Amolops* (Anura: Ranidae) from China, with taxonomic comments on *A. liangshanensis* and Chinese populations of *A. marmoratus*. *Zootaxa*, *4609*(2), 247-268.
251	AB211488.1_A_panhai_Thailand	*Amolops panhai*	AB211488.1	Matsui et al., 2006	Matsui, M., Shimada, T., Liu, W. Z., Maryati, M., Khonsue, W., & Orlov, N. (2006). Phylogenetic relationships of Oriental torrent frogs in the genus *Amolops* and its allies (Amphibia, Anura, Ranidae). *Molecular Phylogenetics and Evolution*, *38*(3), 659-666.

252	JF794451.1_A_panhai_Myanmar	*Amolops panhai*	JF794451.1	Dever et al., 2012	Dever, J. A., Fuiten, A. M., Konu, Ö., & Wilkinson, J. A. (2012). Cryptic torrent frogs of Myanmar: an examination of the *Amolops marmoratus* species complex with the resurrection of *Amolops afghanus* and the identification of a new species. *Ichthyology & Herpetology*, *2012*(1), 57-76.
253	DQ204486.1_A_ricketti_Fujian_China	*Amolops ricketti*	DQ204486.1	Ngo et al., 2006	Ngo, A., Murphy, R., Liu, W., Lathrop, A., & Orlov, N. (2006). The phylogenetic relationships of the Chinese and Vietnamese waterfall frogs of the genus *Amolops*. *Amphibia-reptilia*, *27*(1), 81-92.
254	MK263262.1_A_sinensis_Guandong_China	*Amolops sinensis*	MK263262.1	Lyu et al., 2019	Lyu, Z. T., Zeng, Z. C., Wan, H., Yang, J. H., Li, Y. L., Pang, H., & Wang, Y. Y. (2019). A new species of *Amolops* (Anura: Ranidae) from China, with taxonomic comments on *A. liangshanensis* and Chinese populations of *A. marmoratus*. *Zootaxa*, *4609*(2), 247-268.
255	AF206456.1_A_spinapectoralis_Gia_Lai_Vietnam	*Amolops spinapectoralis*	AF206456.1	Chen et al., 2005	Chen, L., Murphy, R. W., Lathrop, A., Ngo, A., Orlov, N. L., Ho, C. T., & Somorjai, I. L. (2005). Taxonomic chaos in Asian ranid frogs: an initial phylogenetic resolution. *The Herpetological Journal*, *15*(4), 231-243.
256	EF453744.1_A_torrentis_Hainan_China	*Amolops torrentis*	EF453744.1	Cai & Zhang (submitted 2007)	Cai, H., & Zhang, Y. (Unpublished). A Re-analysis of the Phylogeny of the Genus *Amolops* (Anura, Ranidae), with Particular Reference to the Systematic Position of Wurana tormota.
257	MG991904.1_A_tuberodepressus_China	*Amolops tuberodepressus*	MG991904.1	Lyu et al., 2018	Lyu, Z. T., Wu, J., Wang, J., Sung, Y. H., Liu, Z. Y., Zeng, Z. C., ... & Wang, Y. Y. (2018). A new species of *Amolops* (Anura: Ranidae) from southwestern Guangdong, China. *Zootaxa*, *4418*(6), 562-576.
258	AB211480.1_A_viridimaculatus_Yunnan_China	*Amolops viridimaculatus*	AB211480.1	Matsui et al., 2006	Matsui, M., Shimada, T., Liu, W. Z., Maryati, M., Khonsue, W., & Orlov, N. (2006). Phylogenetic relationships of Oriental torrent frogs in the genus *Amolops* and its allies (Amphibia, Anura, Ranidae). *Molecular Phylogenetics and Evolution*, *38*(3), 659-666.
259	KX507304.1_A_wuyiensis_China	*Amolops wuyiensis*	KX507304.1	Sung et al., 2016	Sung, Y. H., Hu, P., Wang, J., Liu, H. J., & Wang, Y. Y. (2016). A new species of *Amolops* (Anura: Ranidae) from southern China. *Zootaxa*, *4170*(3), 525-538.
260	MK263262.1_A_yatseni_China	*Amolops sinensis*	MK263262.1	Lyu et al., 2019	Lyu, Z. T., Huang, L. S., Wang, J., Li, Y. Q., Chen, H. H., Qi, S., & Wang, Y. Y. (2019). Description of two cryptic species of the *Amolopsricketti* group (Anura, Ranidae) from southeastern China. *ZooKeys*, (812), 133.
261	KX507312.1_A_yunkaiensis_China	*Amolops sp. YYW-2016*	KX507312.1	Sung et al., 2016	Sung, Y. H., Hu, P., Wang, J., Liu, H. J., & Wang, Y. Y. (2016). A new species of *Amolops* (Anura: Ranidae) from southern China. *Zootaxa*, *4170*(3), 525-538.
262	MN953751.1_Babina_hainanensis_China	*Nidirana hainanensis*	MN953751.1	Wu et al., 2020	Wu, Y. H., Yan, F., Stuart, B. L., Prendini, E., Suwannapoom, C., Dahn, H. A., ... & Che, J. (2020). A combined approach of mitochondrial DNA and anchored nuclear phylogenomics sheds light on unrecognized diversity, phylogeny, and historical biogeography of the torrent frogs, genus *Amolops* (Anura: Ranidae). *Molecular phylogenetics and Evolution*, *148*, 106789.
263	MN953752.1_Huia_cavitympanum_Malaysia	*Huia cavitympanum*	MN953752.1	Wu et al., 2020	Wu, Y. H., Yan, F., Stuart, B. L., Prendini, E., Suwannapoom, C., Dahn, H. A., ... & Che, J. (2020). A combined approach of mitochondrial DNA and anchored nuclear phylogenomics sheds light on unrecognized diversity, phylogeny, and historical biogeography of the torrent frogs, genus *Amolops* (Anura: Ranidae). *Molecular phylogenetics and Evolution*, *148*, 106789.
264	MN953755.1_Odorrana_jingdongensis_China	*Odorrana jingdongensis*	MN953755.1	Wu et al., 2020	Wu, Y. H., Yan, F., Stuart, B. L., Prendini, E., Suwannapoom, C., Dahn, H. A., ... & Che, J. (2020). A combined approach of mitochondrial DNA and anchored nuclear phylogenomics sheds light on unrecognized diversity, phylogeny, and historical biogeography of the torrent frogs, genus *Amolops* (Anura: Ranidae). *Molecular phylogenetics and Evolution*, *148*, 106789.

265	MN953753.1_Rana_jiemuxiensis_China	*Rana jiemuxiensis*	MN953753.1	Wu et al., 2020	Wu, Y. H., Yan, F., Stuart, B. L., Prendini, E., Suwannapoom, C., Dahn, H. A., ... & Che, J. (2020). A combined approach of mitochondrial DNA and anchored nuclear phylogenomics sheds light on unrecognized diversity, phylogeny, and historical biogeography of the torrent frogs, genus *Amolops* (Anura: Ranidae). *Molecular phylogenetics and Evolution*, *148*, 106789.

Supplementary Table S2. Details of sampling sites surveyed for Amolops chakrataensis in the Chakrata landscape (Dehradun district) and in the Bhagirathi River basin (Uttarkashi district), Uttarakhand, India, between 2023 and 2026. The table summarises geographic location, elevation, survey effort for A. chakrataensis, fieldwork dates and seasonal coverage, habitat characteristics, observed threats, and other amphibian species found at each site.

Sites	Lat.	Long.	Altitude (m a.s.l.)	Number of observers	Number of days of surveys	Dates	Sampling season	Target species	Amphibians sighted	Habitat type	Observed associated threats
Tuna Forest	30.7825	77.8375	1936	2	4	19–25 Jun 2024; 7–10 Oct 2024; 25–28 Apr 2026	II, III, IV	Absent	*Duttaphrynus himalayanus, Nanorana minica, N. vicina*	Deodar–pine forest streams	Plastic pollution
Masak area	30.7698	77.8223	2330	2	2	19–25 Jun 2024; 14–18 Sep 2024	**III, IV**	Absent	*D. himalayanus, N. minica, N. vicina*	Deodar forest streams	Plastic pollution
Jadi village	30.7465	77.8505	2246	3	6	16–21 Feb 2023; 8–11 Apr 2023; 19–25 Jun 2024; 14–18 Sep 2024; 7–10 Oct 2024; 8–13 May 2025; 25–28 Apr 2026	I, II, III, IV	Absent	*N. minica, N. vicina*	Mixed forest–urban interface	Road widening, urbanisation, road widening, plastic pollution, algal blooms
Kanaser	30.7127	77.8662	2029	3	1	8–11 Apr 2023; 19–25 Jun 2024; 14–18 Sep 2024; 7–10 Oct 2024	II, III, IV	Absent	*N. minica, N. vicina*	Deodar forest streams	Plastic pollution
Lokhandi village	30.7586	77.8109	2415	3	4	16–21 Feb 2023; 8–11 Apr 2023; 19–25 Jun 2024; 14–18 Sep 2024	I, II, III, IV	Absent	*N. minica, N. vicina*	Deodar forest streams	Road widening, urbanisation, tourism pressure
Moila forest	30.7765	77.7883	2381	2	3	19–25 Jun 2024; 14–18 Sep 2024; 7–10 Oct 2024; 8–13 May 2025; 25–28 Apr 2026	II, III, IV	Absent	*N. minica, N. vicina*	Deodar–pine forest streams	Household wastewater, plastic pollution
Gvasa forest	30.7229	77.8786	1819	3	3	8–11 Apr 2023; 19–25 Jun 2024; 8–13 May 2025	**II, III**	Absent	*D. himalayanus, Amolops formosus, N. minica*	Forest streams near settlements	Plastic pollution, tourism pressure
Tiger water fall	30.7046	77.894	1524	2	6	16–21 Feb 2023; 8–11 Apr 2023; 19–25 Jun 2024; 14–18 Sep 2024; 7–10 Oct 2024; 8–13 May 2025; 25–28 Apr 2026	I, II, III, IV	Absent	*A. formosus, A. jaunsari, D. himalayanus, N. minica*	Open stream, semi-urban	Tourism pressure, urbanisation
Dava	30.7336	77.8933	1776	2	4	8–11 Apr 2023; 19–25 Jun 2024; 14–18 Sep 2024	II, III, IV	Absent	*D. himalayanus, N. minica*	Agricultural land with oak patches	Agricultural disturbance
Dabri Khadd	30.7701	77.8475	1882	2	2	19–25 Jun 2024; 14–18 Sep 2024; 7–10 Oct 2024	III, IV	Absent	*D. himalayanus*	Agricultural land with oak patches	Agricultural disturbance
Sahiya	30.6241	77.8663	1096	3	4	16–21 Feb 2023; 8–11 Apr 2023; 7–10 Oct 2024	I, II, IV	Absent	*A. jaunsari, D. himalayanus, D. melanosticus, N. minica, Minervarya sp.*	Wide stream/river system	Urbanisation, tourism pressure

Maneri Landscape 1	30.7283	78.5314	1321	2	3	19–23 Oct 2024; 4–6 Jun 2025; 8–14 May 2026	**II, III, IV**	Absent	*A. formosus, A. jaunsari, N. minica*	Stream bordered by forest and paddy fields	Agriculture, dam influence
Maneri Landscape 2	30.7668	78.591	1409	2	5	19–23 Oct 2024; 4–6 Jun 2025; 8–14 May 2026	**II, III, IV**	Absent	*A. formosus, A. jaunsari, N. minica*	Stream bordered by forest and paddy fields	Agriculture, dam influence
Maneri Landscape 3	-	-	1327	2	3	19–23 Oct 2024; 4–6 Jun 2025; 8–14 May 2026	**II, III, IV**	**Present***	*A. chakrataensis*	Stream bordered by forest and paddy fields	Agriculture, dam influence
Maneri Landscape 4	-	-	1435	2	3	19–23 Oct 2024; 4–6 Jun 2025; 8–14 May 2026	**II, III, IV**	Present	*A.chakrataensis, A. formosus, N. minica*	Stream bordered by forest and paddy fields	Agriculture, dam influence

Note: Sampling seasons are defined as I = Spring (February–March), II = Summer (April–May), III = Monsoon (June), and IV = Post-monsoon (September–October). Fieldwork dates indicate periods when field surveys were conducted for amphibians at each locality, whereas the number of survey days represents targeted survey effort for *A. chakrataensis*. *Only a single *A. chakrataensis* individual was captured for buccal swabbing for genetic identification, photography, and morphological measurements. Coordinates are in decimal degrees (WGS84), and elevation is in meters above sea level (m a.s.l.).
